# Fluorene Thiophene α-Cyanostilbene Hexacatenar-Generating LCs with Hexagonal Columnar Phases and Gels with Helical Morphologies as Well as a Light-Emitting LC Display

**DOI:** 10.3390/ijms24119337

**Published:** 2023-05-26

**Authors:** Hongmei Zhao, Xiaohong Cheng

**Affiliations:** 1 Key Laboratory of Medicinal Chemistry for Natural Resource, Ministry of Education, Yunnan Research & Development Center for Natural Products, School of Chemical Science and Technology, Yunnan University, Kunming 650091, China; 2008025@ynau.edu.cn; 2 School of Science, Yunnan Agricultural University, Kunming 650201, China

**Keywords:** fluorene, thiophene-cyanostilbene, polycatenar, self-assembly, liquid crystal, organogel

## Abstract

Two series of novel synthesized hexacatenars, **O/*n*** and **M/*n***, containing two thiophene-cyanostilbene units interconnected by central fluorene units (fluorenone or dicyanovinyl fluorene) using a donor–acceptor–acceptor–donor (A–D–A–D–A) rigid core, with three alkoxy chains at each end, can self-assemble into hexagonal columnar mesophases with wide liquid crystal (LC) ranges and aggregate into organogels with flowerlike and helical cylinder morphologies, as revealed via POM, DSC, XRD and SEM investigation. Furthermore, these compounds were observed to emit yellow luminescence in both solution and solid states which can be adopted to manufacture a light-emitting liquid crystal display (LE-LCD) by doping with commercially available nematic LC.

## 1. Introduction

Polycatenar liquid crystals (LCs) refer to calamitic molecules that are capped at both ends and have three alkyl chains. Such LCs could self-assemble into the columnar phase, meaning they could be applied as organic semiconductors [1,2,3,4]. Further, by introducing functional groups into the rodlike core of polycatenars, nanomaterials with excellent electrooptical characteristics could be achieved [5,6,7,8,9,10,11,12,13,14,15]. 

Conjugated donor–acceptor (D–A) molecules could give rise to efficient fluorescence and charge transport and provide low band gap semiconductors due to intramolecular D–A interactions [16,17,18,19,20,21,22]. As one of the most extensively studied acceptor moieties, fluorene moieties are famous due to their high blue-emitting efficiency and good thermostability [23,24]. Among many donor moieties, thiophene is often used because of its unique optical property and electron transport capability [25,26].

Chirality has great potentials in many areas, such as pharmaceutics [27], asymmetric catalysts [28], chiral-optical devices [29], etc. Chirality could be expressed not only in chiral small molecules, but also in supramolecules such as LCs or organo gels [30,31,32,33,34,35,36,37,38,39,40,41,42]. 

**Scheme 1:**
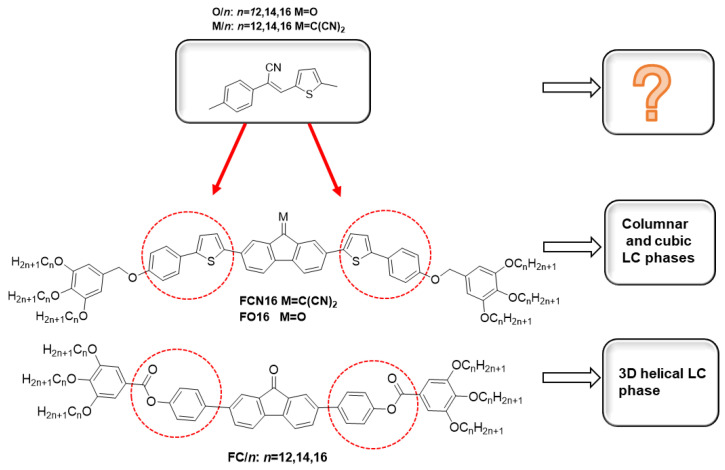
Structures of our previously reported fluorenone-based hexacatenars **FC/*n*** and **FO16** and FCN16, as well as fluorene thiophene α-cyanostilbene hexacatenars **O/*n*** and **M/*n***, that are under investigation.

Controlling the self-assemble behaviors as well as the nano/microstructures of fluorene derivatives is essential for the device performance [43,44,45,46]. Recently, we realized fluorenone-based hexacatenars **FC/*n***, which consist of a short 2,7-diphenyl-9-fluorenone core, displaying columnar and cubic LC phases as well as organo-gels with different morphologies [47,48,49]. Further, we have recently reported that achiral fluorene (either fluorenone or dicyanovinyl fluorene) thiophene contained polycatenars **FO16** and **FCN16**, displaying an interesting 3D helical LC phase which allowed for efficient π–π stacking and displayed interesting ferroelectricity [47,48,49]. Continuing our studies, within this context we have decided to construct novel fluorene thiophene-cyanostilbene based hexacatenars **O/*n*** and **M/*n*** by introducing a α-cyanostilbene (CS) unit which has remarkable electrooptical properties [50,51,52,53,54] into the rigid core of **FO16** and **FCN16** (Scheme 1). The introduction of CS conferred the core A–D–A–D–A characteristics and elongated the rigid core efficiently. The self-assembly and photophysical characteristics and application as emissive devices of these novel polycatenars **O/*n*** and **M/*n*** have been studied. 

## 2. Results and Discussion

### 2.1. Synthesis

**Scheme 2:**
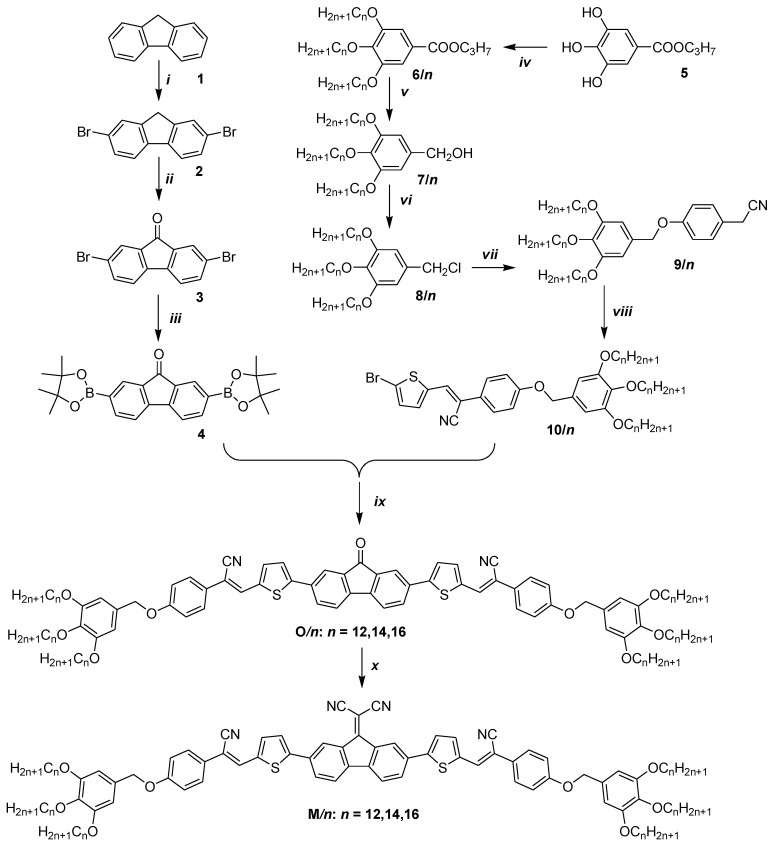
Synthesis of compounds **O*/n*** and **M/*n***. Reagents and conditions: (***i***) Br_2_, FeCl_3_, CHCl_3_, 0 °C, 4 h; (*ii*) CH_3_COOH, CrO_3_, RT, 24 h; (*iii*) bis(pinacolato) diboron, CH_3_COOK, PdCl_2_(dppf), 1,4-dioxane, N_2_, 100 °C, 12 h; (*iv*) C_n_H_2n+1_Br, K_2_CO_3_, DMF, 90 °C, 12 h; (*v*) LiAlH_4_, THF, r.t., 3h; (*vi*) SOCl_2_, THF, 0 °C, 1 h; (*vii*) 4-Hydroxybenzeneacetonitrile, K_2_CO_3_, CH_3_CN, 75 °C, 12 h; (*viii*) 5-bromothiophene-2-carbaldehyde, CH_3_ONa, EtOH, 81 °C, 12 h, (*ix*) Pd(PPh_3_)_4_, K_2_CO_3_, THF, H_2_O, N_2_, 75 °C, 24 h; (*x*) CH_2_(CN)_2_, pyridine, 80 °C, 2 h. (Scheme S1, Figures S12–S28).

Final products **O/*n*** and **M/*n*** were prepared according to the method of synthesis shown in Scheme 2. Firstly, fluorene was bromided with bromine in chloroform yielding 2,7-dibromofluorene, followed by oxidation with chromium trioxide, producing 2,7-dibromofluorenone **3** [55,56]. Then, compound **3** was further reacted with bis(pinacolato) diboron, leading to 2,7-fluorenone borate **4**. 4-hydroxyphenylacetonitrile was etherified with benzyl chloride **8/*n*** [57] to afford intermediate **9/*n***. A Knoevenagel reaction between intermediate **9/*n*** and 5-bromo-2-thiophene carbaldehyde produced intermediate **10/*n***. A Suzuki coupling reaction between **10/*n*** and **4** generated the target compounds **O/*n***. **O/*n*** were converted to **M/*n*** via a condensation reaction with malononitrile. The synthesis methods and structural identification data are displayed in Supporting Information.

### 2.2. Mesomorphic Properties

**Figure 1:**
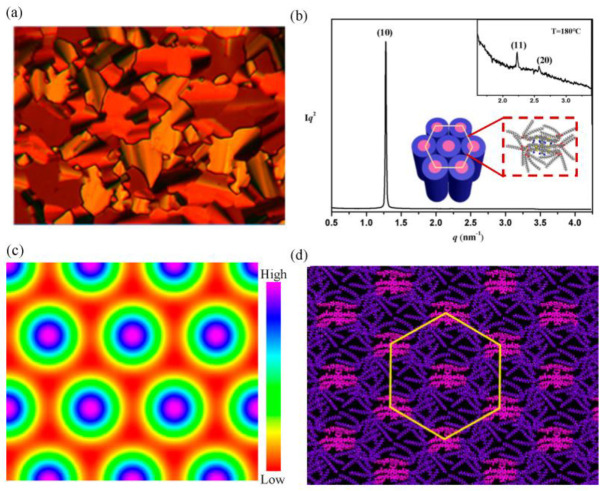
(**a**) The Col_hex_/*p*6*mm* phase of **M/14** cooling from isotropic states at 200 °C; (**b**) SAXS diffraction patterns of the Col_hex_/*p*6*mm* phase of compound **M/14** recorded at 180 °C; the inserts are the suggested molecular packing of the Col_hex_/*p6mm* phase and the molecules array in a disk; (**c**) the electron density map of **M/14**; purple and blue = fluorenone malononitrile central cores, green = aromatic rigid cores, red = aliphatic chains; (**d**) snapshot of molecular dynamic simulation for the Col_hex_/*p*6*mm* phase.

**Figure 2:**
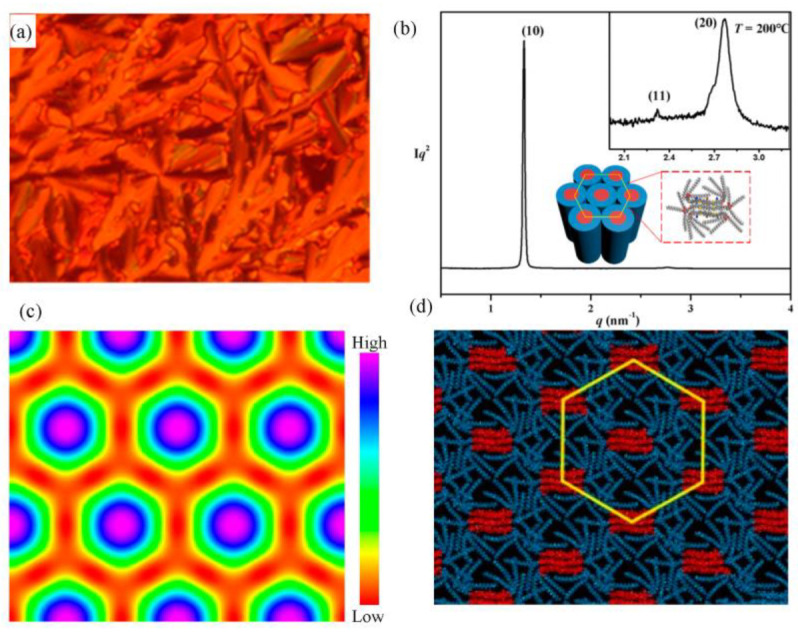
(**a**) The Col_hex_/*p*6*mm* phase of **O/12** cooling from isotropic states at 100 °C; (**b**) SAXS diffraction patterns of the Col_hex_/*p*6*mm* phase of compound **O/12** recorded at 180 °C; the inserts are the suggested molecular packing of the Col_hex_/*p*6*mm* phase and the CPK molecular model of the molecular arrangement in a circular disk; (**c**) the electron density map of **O/12**; purple and blue = fluorenone central cores; green = aromatic rigid cores; red = aliphatic chains; (**d**) snapshot of the molecular dynamic simulation for the Col_hex_/*p*6*mm* phase.

Polarized optical microscopy (POM), differential scanning calorimetry (DSC) and X-ray diffraction (XRD) were used to investigate the LC properties of the synthesized compounds **O/*n*** and **M/*n*** (Table 1). Under POM, compounds **O/*n*** and **M/*n*** exhibited mosaic and spherulitic textures as specialized for columnar phases in their LC ranges (Figure 1a, Figure 2a and Figure S1). The small angle X-ray diffraction (SAXS) pattern of **M/14** at 180 °C (Figure 1b) showed d^−2^ values of the three peaks in a ratio of 1:3:4, with the respective Miller indices, (10), (11) and (20), indicating a two-dimensional hexagonal lattice. The lattice constant of **M/14** calculated using the XRD results was 5.65 nm (Table 1 and Table S1). The number of molecules in the cross section of column was calculated to be about three (Table 1). To satisfy the maximum amount of space filling, three molecules should be parallelly aligned in a disc. Afterwards, these discs were stacked into a cylinder, and the formed cylinders further arranged into a columnar liquid crystal phase with a *p*6*mm* lattice (Figure 1b, insert map). Micro-segregation of the central rigid aromatic core from the peripheral flexible alkyl chains and π–π stacking are the driving forces for such columnar packing. A snapshot of the molecular dynamic simulation for the Col_hex_/*p*6*mm* phase (Figure 1d) as well as the reconstructed ED map (Figure 1c) also demonstrated such packing.

The SAXS pattern for **O/12** at 200 °C is shown in Figure 2b, the d^−2^ values of the three peaks in the ratio of 1:3:4, which can also be indexed to the 10, 11 and 20 reflections of hexagonal lattice with *p*6*mm* symmetry. The lattice constant of **O/12** calculated using the XRD results is 5.37 nm (Table 1 and Table S2). The number of molecules (*μ*) in the cross section of the columns was approximated as three (Table 1, Table S3 and S4). The suggested packing model for such a hexagonal columnar phase is shown in the insert of Figure 2b, which is in line with the molecular dynamics (MD) annealed model (Figure 2d) as well as the reconstructed electron density map (Figure 2c).

Since only (10) and (11) diffraction peaks in the SAX pattern of the column phase are observed for the **O/16** (Figure S6), a boundary experiment was carried out with **M/14** (Figure S1e). The uninterrupted developing spherulitic texture of the Col_hex_/*p*6*mm* phase of compound **M/14** into the area of **O/16** without any visible miscibility gap (Figure S1e) demonstrated that the columnar phase of **O/16** was a hexagonal columnar phase similar to that of **M/14**. Spherulitic textures with dark areas were also displayed by the fluorene dicyanovinylene-based polycatenar **M/16** with longest alkyl chains under POM (Figure S1d). Binary mixtures with **M/14** demonstrated that the columnar phase of **M/16** was also a hexagonal columnar phase (Figure S1f).

The optimized molecular structures of **O/*n*** and **M/*n***, calculated using density functional theory (DFT) (Gaussian 09W B3LYP/(6–31G, d)), are shown in Figure S11. It can be seen that the two cyano groups from CS, the carbonyl group of fluorenone and the cyano groups of fluorenone malononitrile are located on the same side of the π-conjugated rigid core. 

**Figure 3:**
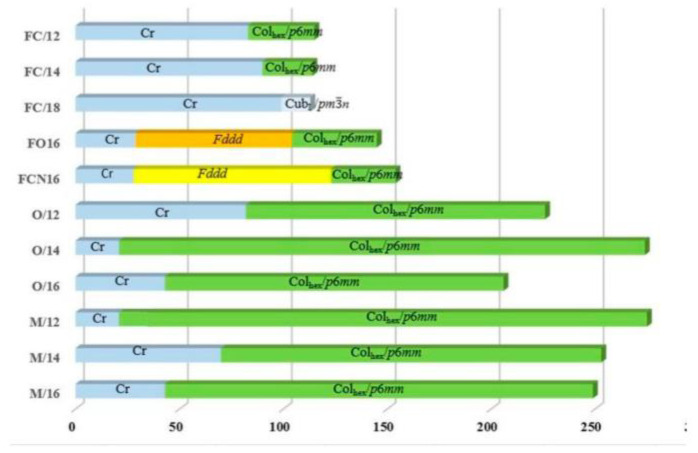
The molecular structures of the previously reported analogues **FC/*n***, **FO16** and **FCN16** and compounds **O/*n*** and **M/*n*** under investigation, as well as the bar graph-summarized phase transitions of these compounds.

By comparison with the former reported fluorene polycatenars **FC/*n*** [47], **FO16** and **FCN16** [48] reported here have compounds with broader LC ranges (Figure 3); the introduction of thiophene α- increases greatly increases greatly the clear points, and the mesophase ranges are also widened greatly. Clearly, this should be due to the extended conjugated units reinforcing the π–π stacking, with the introduction of α-cyanostilbene group producing a strong dipole–dipole interaction.

### 2.3. Gelation Behavior

Compounds **O/16** and **M/14** were chosen as representatives to study the gel properties of these compounds. Investigation with “stable to inversion of the container” methods [58] indicated that both **O/16** and **M/14** could generate organogels in 1,4-dioxane (10 mg/mL) (Table 2).

**Figure 4:**
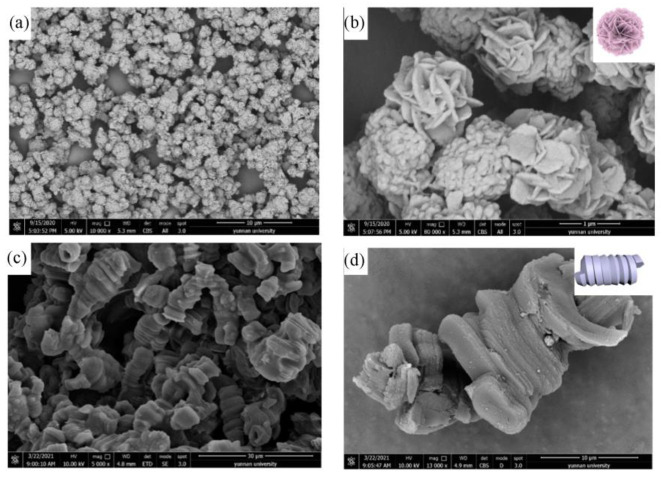
Selected SEM images of xerogels formed in1,4-dioxane: (**a**) **O/16**, scale bar is 10 μm; (**b**) partially enlarged view of (**a**), scale bar is 1 μm; (**c**) **M/14**, scale bar is 30 μm; (d) partially enlarged view of (**c**), scale bar is 10 μm.

The morphologies of the xerogels were studied via SEM investigation. Xerogel formed by **O/16** in 1,4-dioxane displayed a microsphere with flower-like morphologies. The sizes of the spheres were non-uniform, with the diameter ranging from 1 μm to 2.72 μm (Figure 4a,b). The enlarged image of an individual sphere indicated that the spheres were constituted by a nanosheet with a thickness from 30 nm to 100 nm and an average diameter of 0.4–0.6 μm, as shown in Figure 4b. Interestingly, the gel morphology of **M/14** in 1,4-dioxane showed the typical spiral cylindrical morphologies, with diameters of about 3.75–12.5 μm (Figure 4c,d). The dipolar–dipolar interaction, π–π interaction, Var der Waals force, etc., could all promote the formation of gels. The possible gel formation process is demonstrated in SI (Figure S7).

### 2.4. Photophysical Properties

**Figure 5:**
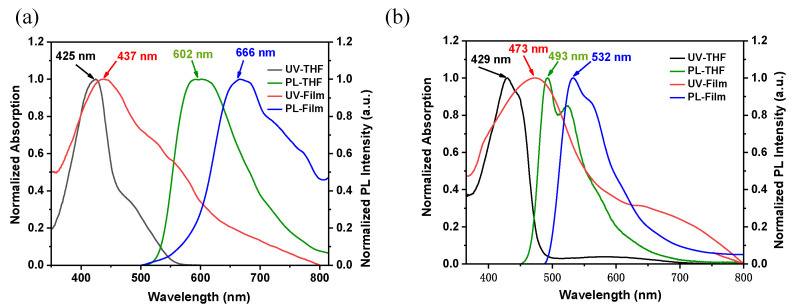
Normalized absorption and emission spectra of (**a**) **O/12** (the UV-vis absorption and excitation wavelengths of **O/12** in THF are 425 nm and 602 nm, and with the thin film they are 437 nm and 666 nm); (**b**) **M/12** (the UV-vis absorption and excitation wavelengths of **M/12** in THF are 429 nm and 493 nm, and with the thin film they are 473 nm and 532 nm) (1.0 × 10^−5^ mol L^−1^).

The UV-vis and fluorescence spectra of the representative compounds **O/12** and **M/12** were investigated (Table 3, Figure 5 and Figure S8). Compared with the UV-vis spectra in the solution, the UV-vis spectra of **O/12** and **M/14** in the thin film displayed red-shifts from 425 to 437 nm and 429 to 473 nm, respectively (Table 3, Figure 5), and the fluorescence spectra of the films were also red-shifted (602 to 666 nm for **O/12** and 493 to 532 nm for **M/12**). Thus, a J-type parallel π–π aggregating style in the solid was suggested [59]. The emission spectra of **O/12** in the dilute solution and solid state displayed emissions at 602 nm and 666 nm, respectively. This means a large Stokes shift (177 nm in the THF solution, and 229 nm in solid state). The energy gap calculated using the UV spectra $E_{g}^{\mathrm{opt}}$ (eV), DFT calculations (B3LYP/6-31G) $E_{g}^{\mathrm{cal}}$ (eV) and cyclic voltammetry $E_{g}^{\mathrm{CV}}$ (eV) (Figures S9 and S10) are almost identical, with the value about 2.60 eV for **O/12** and 2.20 eV for **M/12**. The energy gap of **M/*n*** is smaller than that of O/*n*; this may be due to the stronger electron drawing of malononitrile in **M/*n***. This investigation indicated that these compounds had potential as semiconductors.

The solvent effects on the photophysical properties of representative compound **O/12** were studied. The absorption spectra changed little by increase of the solvent polarity, meaning that the solvent polarity had little influence on the structure and electronic properties of the ground state (Figure S14a), while the fluorescence spectra changed largely with increasing solvent polarity (Figure S14b). Fluorescence quantum yields (*Φ*_FL_) measured in different solutions ranged from 0.061 to 0.579 (Table 4) [60]. The *Φ*_FL_ of **O/12** was 0.251 in solid state, indicating that **O/12** could be suitable for LE-LCD and bioimage application (Table 4) [61,62,63,64,65], while the fluorescence of **M/14** was very weak in both the solution and solid state, which is unsuitable for LE-LCD studies (Figure S8).

Surprisingly, the AIEE phenomena usually observed in CS compounds are only observed for intermediate **10/12**, but not for the final product **O/12**; the corresponding experimental data and explanation are shown in SI (Figures S12 and S13).

### 2.5. Polarized Emission Spectra, the Dichroic ratio and LE-LCD Device

**Figure 6:**
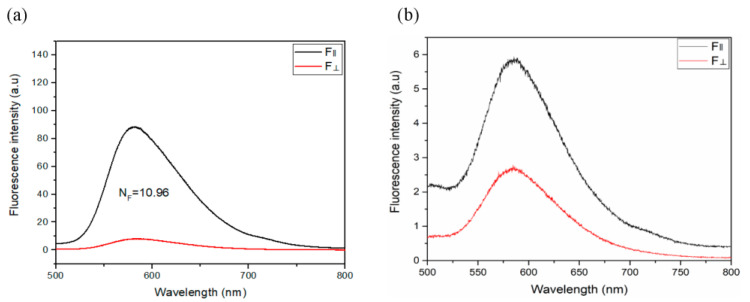
Polarized fluorescence spectra of light-emitting LC cell of 0.5 wt% **O/12** + Nematic LC SLC9023 with 1 KHz, 8 V; (**a**) when electric field is turned off; (**b**) when electric field is turned on. The dichroic ratio (N_F_) was calculated according to the formula N_F_ = F_//_/F_┴_, in which F_┴_ and F_//_ are the rubbing direction of the LC cell parallel and perpendicular to the polarization direction of the detector, respectively.

The solid fluorescence quantum yield of compound **O/12** was measured to be 25.1%. Thus, the potential application of **O/*n*** as an LE-LCD device was studied. The dichroic ratio of the polarized fluorescence spectra for the LC mixture of 0.5 wt% **O/12** with nematic LC SLC9023 was measured to be 10.96 when the electric field was off (Figure 6a), and about 2.17 when the electric field was on (Figure 6b). Such values mean that the LC mixture of **O/12** and SLC9023 were suitable for fabrication of the LE-LCD device. 

**Figure 7:**
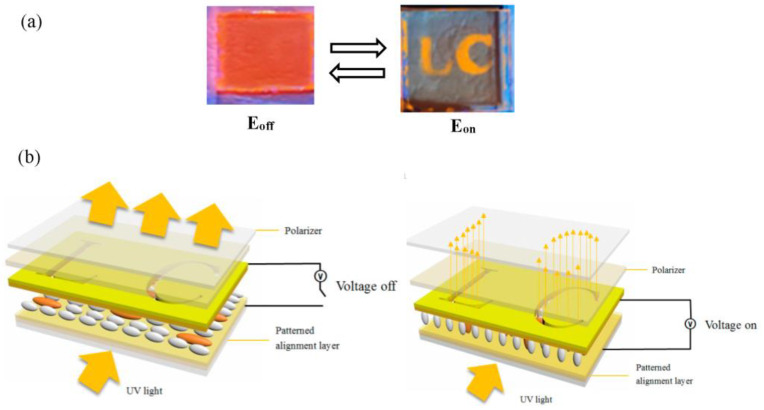
(**a**) Photograph of the LE-LCD device in the electric field-off and field-on states using the light-emitting LC mixture (Nematic LC SLC9023 + 0.5 wt% **O/12**); (**b**) the LE-LCD device with patterned alignment in the electric field-off and field-on states using the LC mixture.

Thus, the LE-LCD device was homemade. Firstly, patterned ITO glass substrates were used to prepare the LC cell. Then, LC mixtures (SLC9023 + 0.5 wt% **O/12**) were poured into the LC cell. The unidirectional array of the LC mixtures was obtained by rubbing the polyimide (PI)-aligned layer (the device fabrication details are presented in SI). The LC mixtures were irradiated using a UV lamp. The variation in PL efficiency was detected using a polarizer, the transmission direction of which was transmitted parallel to the LC-aligned direction. When the electric field was turned off, the LC cell under UV illumination was bright. Under an electric field of 1 KHz and 5 v, only the central region without ITO was bright. Thus, switching from brightness to darkness was realized (Figure 7).

## 3. Conclusions

Two novel series of A–D–A–D–A π-conjugated hexacatenars, consisting of a fluorenone or dicyanoethenyl fluorene core and with thiophene-cyanostilbene on both sides, can self-organize into columnar phases with a *p*6*mm* lattice and form organogels with nanoflower and helical cylinder morphologies. Finally, the potential of **O/12** as a yellow-emitting LE-LCD device has been realized. Thus, the design principle which combines central fluornene with thiophene-cyanostilbene on both sides of the rigid core is a successful strategy to obtain stable columnar mesophases with wide LC ranges, orgnogels with interesting chiral morphologies and semiconductor materials with interesting properties. 

## Figures and Tables

**Table 1:** Phase transition temperatures of **O/*n*** and **M/*n****^a^*.

Comp.	*n*	*T*/°C [∆*H*/kJ·mol^−1^]	*a/*nm (T/°C*)*	*μ*
**O/12**	12	Cr 82 Col_hex_/*p*6*mm* 226 Iso (Iso 222 Col_hex_/*p*6*mm* 73 Cr)*^c^*	5.37 (200)	3.44
**O/14**	14	Cr 21 [28.84] Col_hex_/*p*6*mm* 238 [2.07] Iso (Iso 233 [2.08] Col_hex_/*p*6*mm* 11 [28.17] Cr)	5.65 (180)	3.44
**O/16**	16	Cr 43 [65.61] Col_hex_/*p*6*mm* 206 [1.09] Iso (Iso 206 [1.03] Col_hex_/*p*6*mm* 28 [53.00] Cr)	6 (180)	3.16
**M/12**	12	Cr20*^c^* Col_hex_/*p*6*mm^b^* 275 [0.89] Iso (Iso 259 [0.60] Col_hex_/*p*6*mm* Cr20)*^c^*	-	-
**M/14**	14	Cr 70*^c^* Col_hex_/*p*6*mm* 253 [2.62] Iso (Iso 230 [1.39] Col_hex_/*p*6*mm* 68*^c^* Cr)	5.65 (180)	3.36
**M/16**	16	Cr 43 [22.65] Col_hex_/*p*6*mm ^b^* 249 [2.21] Iso (Iso 243 [0.96] Col_hex_/*p*6*mm*17 [12.78] Cr)	-	-

*^a^* Date from DSC second scan, 5 K min^−1^ (Figure S2). Cr = crystalline solid; Iso = isotropic liquid; Col_hex_/*p*6mm = hexagonal columnar phase; *a* = lattice parameter determined via SAXS; *μ* = number of molecules in the cross section of the column (*μ* = (a^2^/2)⎷3*h*(*N_A_*/*M*)*ρ*, presuming density of *ρ* = 1 g/cm^3^ and a columnar disk height of *h* = 0.43–0.44 nm equal to the measured maxima of the diffuse wide-angle scatterings, as displayed in Figures S3, S4, S5b and S6b). *^b^* was identified via contact experiment, which showed that all compounds are of a hexagonal columnar phase. *^c^* Transition temperatures were identified via POM.

**Table 2:** Gel test of compounds **O/16** and **M/14** ^a^.

Solvent	O/16	M/14
Ethyl acetate	P	P
Acetone	P	P
n-hexane	P	P
Toluene	S	S
Acetonitrile	P	P
t-butanol	S	S
Dichloromethane	S	S
Petroleum ether	S	S
1,4-Dioxane	G	G
Ethanol	P	P

^a^ S: soluble; G: gelation; P: precipitation.

**Table 3:** Absorption and emission data of compounds **O/12** and **M/12** in the THF solution and solid state.

Compd.	Abs λ_max_(nm)	PL *^a^* λ_max_ (nm)	Stokes Shift *^b^*(nm)	$E_{\mathrm{onset}}^{\mathrm{red}}$ (V)	$E_{\mathrm{onset}}^{\mathrm{ox}}$ (V)	*E*_HOMO_ (eV)	*E*_LUMO_ (eV)	$E_{g}^{\mathrm{opt}}$ (eV)	$E_{g}^{\mathrm{cal}}$ (eV)	$E_{g}^{\mathrm{CV}}$ (eV)
**O/12**	425 *^c^* (437) *^d^*	602 *^c^* (666) *^d^*	177 *^c^* (229)*^d^*	−0.70	1.90	−5.32	−2.73	2.75	2.59	2.60
**M/12**	429 *^c^* (473) *^d^*	493 *^c^* (532) *^d^*	64 *^c^* (59) *^d^*	−0.70	1.45	−5.39	−3.41	2.20	1.98	2.20

*^a^* Excited with absorption maxima band; *^b^* Stokes shifts: λ_em_–λ_Abs_; *^c^*measured in the THF solution; *^d^*measured in solid state; relative to quinine sulfate in 0.1 M H_2_SO_4_ (*Φ*_FL_ = 0.54) as the standard; $E_{g}^{\mathrm{OPT}}$ energy band gap in THF solution calculated using the UV-vis spectrum; according to *hν* − (A*hν*)^2^, the tangent of the curve edge is made, and the intersection with the X axis is the spectral energy gap of the compound; $E_{g}^{\mathrm{CV}}$ genergy band gap in film calculated using CV on glassy carbon electrode in 0.1 mol/L of Bu_4_NBF_4_ in CH_3_CN with a scan rate of 100 mVs^−1^, *E*_g_^2^ = *E*_ox_ − *E*_red_; *E*_g_^cal^ energy band calculated using DFT/B3LYP, 6–31G (**d**), *E*_g_^cal^ = *E*_LUMO_ − *E*_HOMO_.

**Table 4:** Absorption, emission and fluorescence quantum yield of compound **O/12** at RT.

Solvents	λ_abs_(nm)	λ_em_(nm) *^a^*	Stokes Shift (nm) *^b^*	*Φ*_FL_ *^c^*
Hexane	420	655	235	0.061
Toluene	424	582	158	0.579
DCM	425	637	212	0.114
THF	425	602	177	0.484
1,4-Dioxane	421	592	171	0.40
solid state	-	-	-	0.251

*^a^* Excited at absorption maxima band. *^b^* Stokes shifts = λ_em_ − λ_abs_. *^c^*Relative to fluorescein in 0.1 M H_2_SO_4_ (*Φ*_FL_ = 0.54) as the standard.

## Data Availability

Data are contained within the article.

## Supplementary Materials

The following supporting information can be downloaded at: https://www.mdpi.com/article/10.3390/ijms24119337/s1. References [66,67,68,69,70,71,72,73] are cited in the Supplementary Materials.
