# Supplementary material for: Fluorene Thiophene α-Cyanostilbene Hexacatenar-Generating LCs with Hexagonal Columnar Phases and Gels with Helical Morphologies as Well as a Light-Emitting LC Display
Source: Int J Mol Sci. 2023 May 26;24(11):9337. doi: 10.3390/ijms24119337 (PMC10253829; doi:10.3390/ijms24119337)
Supplement: Supplementary file 1 [file ijms-24-09337-s001.zip › ijms-2401083-supplementary.pdf]

Supporting information

Fluorene Thiophene α-Cyanostilbene Hexacatenar-Generating LCs with Hexagonal Columnar Phases and Gels with Helical Morphologies as well as a Light-Emitting LC Display

Hongmei Zhao [1,2] and Xiaohong Cheng [1,*]

[1] Key Laboratory of Medicinal Chemistry for Natural Resource, Ministry of Education, Yunnan Research & Development Center for Natural Products, School of Chemical Science and Technology, Yunnan University, Kunming 650091, China; 2008025@ynau.edu.cn

[2] School of Science, Yunnan Agricultural University, Kunming 650201, China

Content

1. Additional Experimental Data S02

2. Materials, synthesis and analytical data S11

3. References S22

1 Additional Experimental Data

1.1 Experimental techniques

A Mettler heating stage (FP 82 HT) was used for polarizing optical microscopy (POM, Optiphot 2, Nikon) and DSC were recorded with a DSC 200 F3 Maia calorimeter (NETZSCH) at 10 K min^{-1}. The UV-vis absorption and fluorescence spectra were carried out on UV2600A UV-vis absorption spectrometer (UNICO, China).

SEM experiments were carried out on a QUNT200 scanning electron microscopy (SEM, USA). All pictures were taken digitally. For the sample preparation, the gel was placed on an aluminium foil for some time until the gel became dry gel, then the sample was gold plated, finally the sample was put the sample into the scanning electron microscopy for observation. XRD measurement of xerogels was used the X-ray powder diffraction (XRD, Rigaku Co., Tokyo, Japan) analysis was conducted on a D/max-3B spectrometer with Cu Kα radiation.

Small-angle powder diffraction (SAXS) experiments were performed in transmission mode with synchrotron radiation at the 1W2A SAXS beamline at Beijing Accelerator Laboratory[S1]. A modified Linkam hot stage with a thermal stability within 0.2 °C was used, with a hole for the capillary drilled through the silver heating block and mica windows attached to it on each side. Samples were held in the poly(imide) (Kapton) film. A MarCCD 165 detector was used. q calibration and linearization were verified using several orders of layer reflections from silver behemate. Positions and intensities of the diffraction peaks were measured using PeakSolve™ (Galactic).

Electron density reconstruction. Fourier reconstruction of the electron density was carried out using the general formula for 2D periodic systems:

$E(xy) = \Sigma_{hk}\ \mathrm{sqrt}[I(hk)]\ \exp[i2\pi(hx+ky) + \phi_{hk}]$

here m being the multiplicity, F the structure factor, which is proportional to the intensity and φ the phase of the reflex.

SLC9023 (Δn = 0.251 (589 nm, 20 °C), $\Delta\varepsilon$ = 1.517 (1 kHz, 20 °C)) was purchased from Slichem Co., Ltd, China, The planar oriented LC cells (cell gap: 4.8 μm) with a homogeneously rubbed polyimide (PI) alignment layer were purchased from SOOBOO INTL SHARES LIMETED (China). The LC mixtures were prepared by combining solutions of the SLC9023 and 0.2wt% of **O/12** in dichloromethane. The resulting solutions were then sonicated for about 1 min in order to achieve good solution, and thereafter, dichloromethane was evaporated off completely. Then the LC mixture was filled into the empty cell by capillary action. The polarized emission spectra of the mixture in LC cell were measured by Hitachi F-7000 fluorescence spectrometer (Hitachi, Japan) with a polarization unit (P/N 250-2420). The rubbing direction of the LC cell must be made sure to be parallel to the analyzer. After we get the fluorescence intensity for parallel

irradiation($F_{//}$) and the fluorescence intensity for perpendicular irradiation ($F_{\perp}$), the dichroic ratio (N_F) was determined from the formula: $N_F = F_{//}/F_{\perp}$". For preparation of the LE-LCD device, commercially available LC cell coated with both ITO and the polyimide (PI) alignment layer was etched firstly. Thus such LC-cell was splitted into half. The surface coated with ITO and PI was covered with a mask with the hollow letters "LC". The ITO and PI in the exposed area of the letters were rubbed off with cotton which has been soaked with 50% hydrochloric acid and dipped some zinc powder, so that the letters were imprinted on the glass. The exposed area was cleared with water; then the mask was removed off. The edge of the glass was coated with B-7000 glue, and pressed with another unetched half piece of glass into one LC cell again for LC mixture filling.

The quantum yields of compounds **O/12** were determined using quinolinium hydrogen sulphate in H_2SO_4 (Φ_{FL}=0.55) as standard and applying the following equation[S2].

$$\Phi F_{sa} = \Phi F_{st} \frac{A_{st} * I_{sa} * n_{sa}^2}{A_{sa} * I_{st} * n_{st}^2}$$

sa = sample; *st* = standard (quinolinium hydrogen sulphate in H_2SO_4); A = absorbance; I = integration of corrected fluorescence spectrum; *n* = refractive index of the solvent.

1.2 Additional textures of LC phases and DSC traces

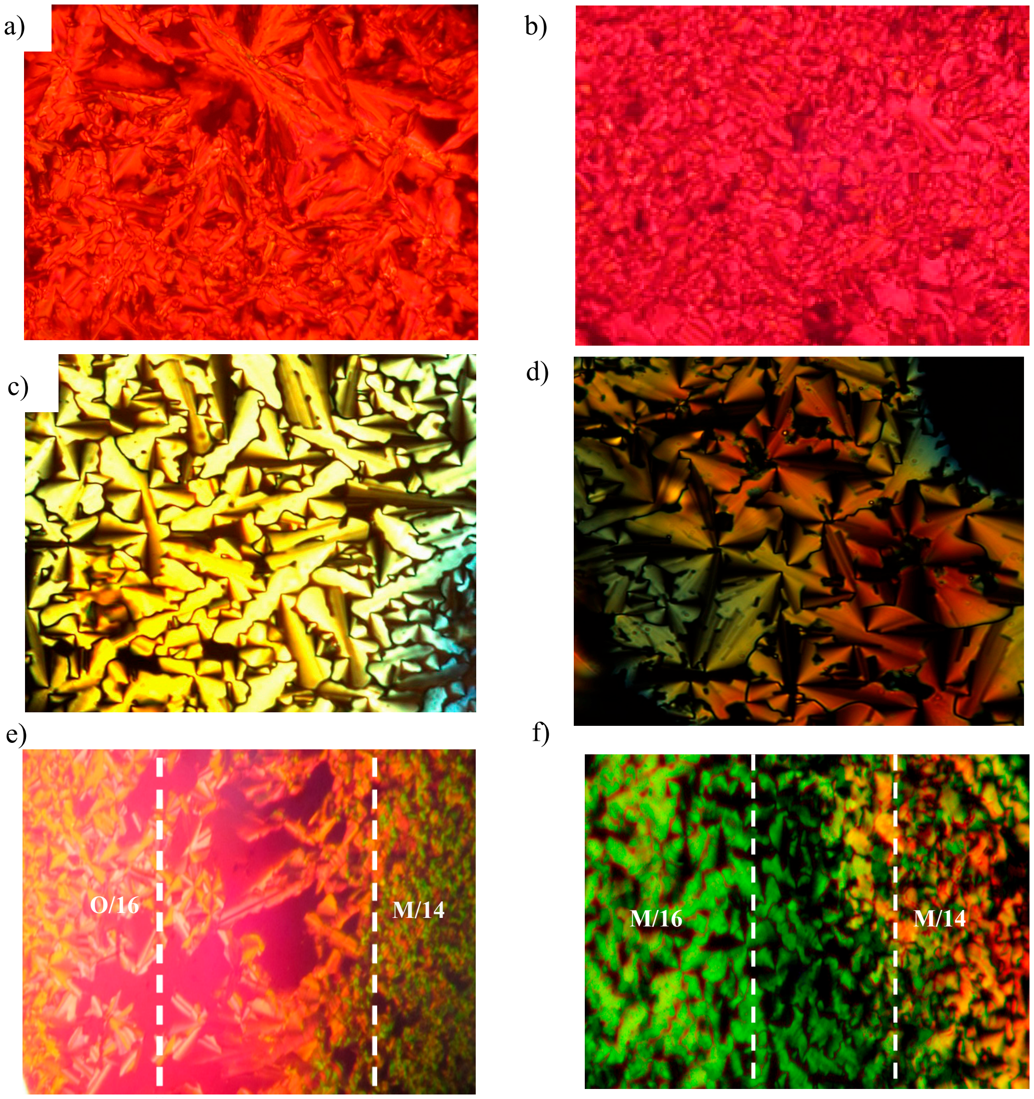

Figure S1. Representative textures between crossed polarizers: a) Col_{hex}/*p*6*mm* phase of **O/14** cooling at 150 °C; b) Col_{hex}/*p*6*mm* phase of **O/16** cooling at 150 °C; c) Col_{hex}/*p*6*mm* phase of **M/12** cooling at 200 °C; d) Col_{hex}/*p*6*mm* phase of **M/16** cooling at 200 °C; e) Contact region between the LC phases of **O/16** and **M/14** at T = 100 °C, dotted line indicate the approximate position of the boundary between the two compounds; f) Contact region between the LC phases of **M/16** and **M/14** at T = 150 °C, dotted line indicate the approximate position of the boundary between the two compounds.

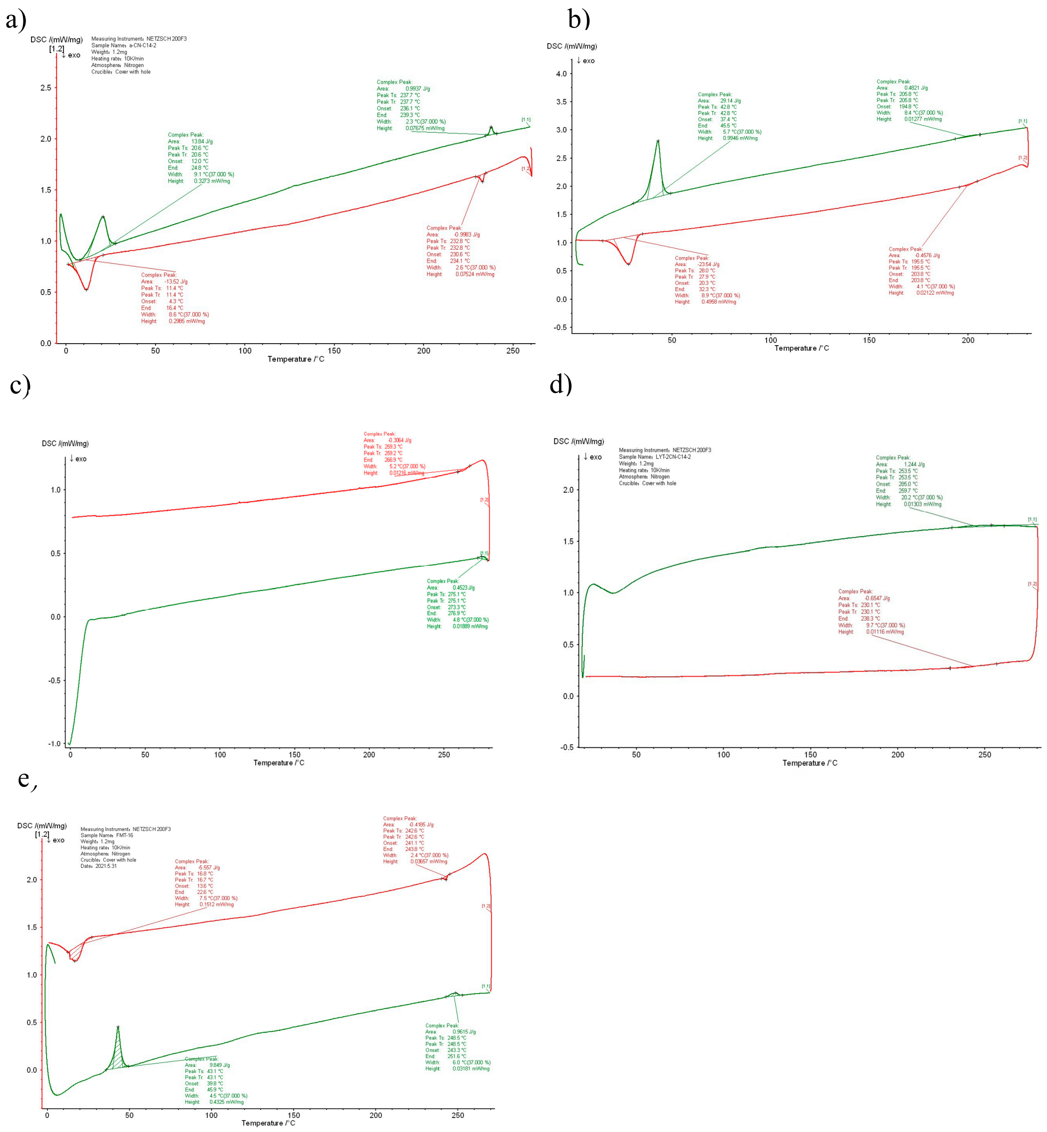

Figure S2. a) DSC heating and cooling scans (5 K min^{-1}) of compound **O/14**; b) DSC heating and cooling scans (5 K min^{-1}) of compound **O/16**; c) DSC heating and cooling scans (5 K min^{-1}) of compound **M/12;** d) DSC heating and cooling scans (10 K min^{-1}) of compound **M/14**; e) DSC heating and cooling scans (10 K min^{-1}) of compound **M/16.**

Table S1. Experimental and calculated *d*-spacings of the observed SAXS reflections of the $Col_{hex}/p6mm$ phase in compound **M/14** at 180 °C. All intensity values are Lorentz and multiplicity corrected.

(*hk*)	$d_{obs.}$–spacing (nm)	$d_{cal.}$–spacing (nm)	Intensity	*Phase*
(10)	4.92	4.89	100	0
(11)	2.81	2.83	0.09	0
(20)	2.45	2.45	0.03	π
a_{hex} = 5.65 nm				

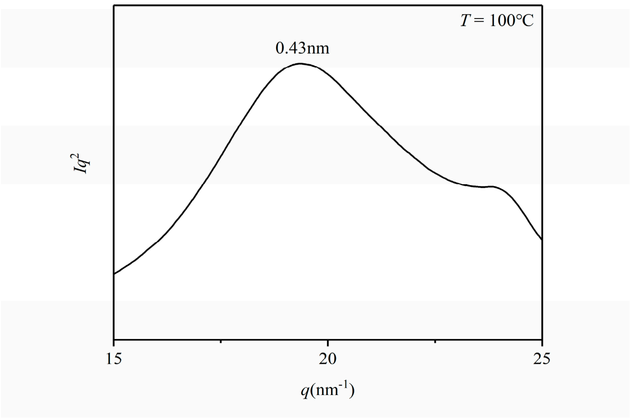

Figure S3. WAXS diffraction patterns of the Col_{hex}/*p*6*mm* phase of compound **M/14** recorded at 100 °C.

Table S2. Experimental and calculated *d*-spacings of the observed SAXS reflections in compound **O/12** at 200 °C.

(*hk*)	$d_{obs.}$–spacing (nm)	$d_{cal.}$–spacing (nm)	Intensity	*Phase*
(10)	4.72	4.65	100	0
(11)	2.71	2.69	0.05	0
(20)	2.27	2.32	2.14	π
a_{hex} = 5.37 nm				

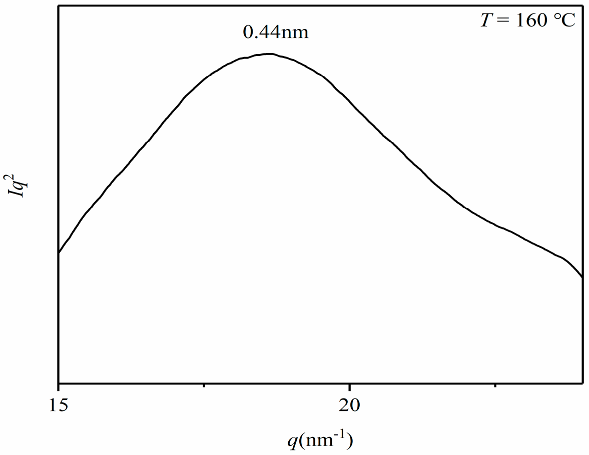

Figure S4. a) WAXS diffraction patterns the Col_{hex}/*p*6*mm* phase of compound **O/12** recorded at 160 °C.

Table S3. Experimental and calculated *d*-spacings of the observed SAXS reflections in compound **O/14** at 180 °C.

(*hk*)	$d_{obs.}$–spacing (nm)	$d_{cal.}$–spacing (nm)	Intensity	*Phase*
(10)	4.89	4.87	100	0
(11)	2.81	2.82	0.04	0

(20)	2.43	2.43	0.03	π
a_{hex} = 5.65 nm				

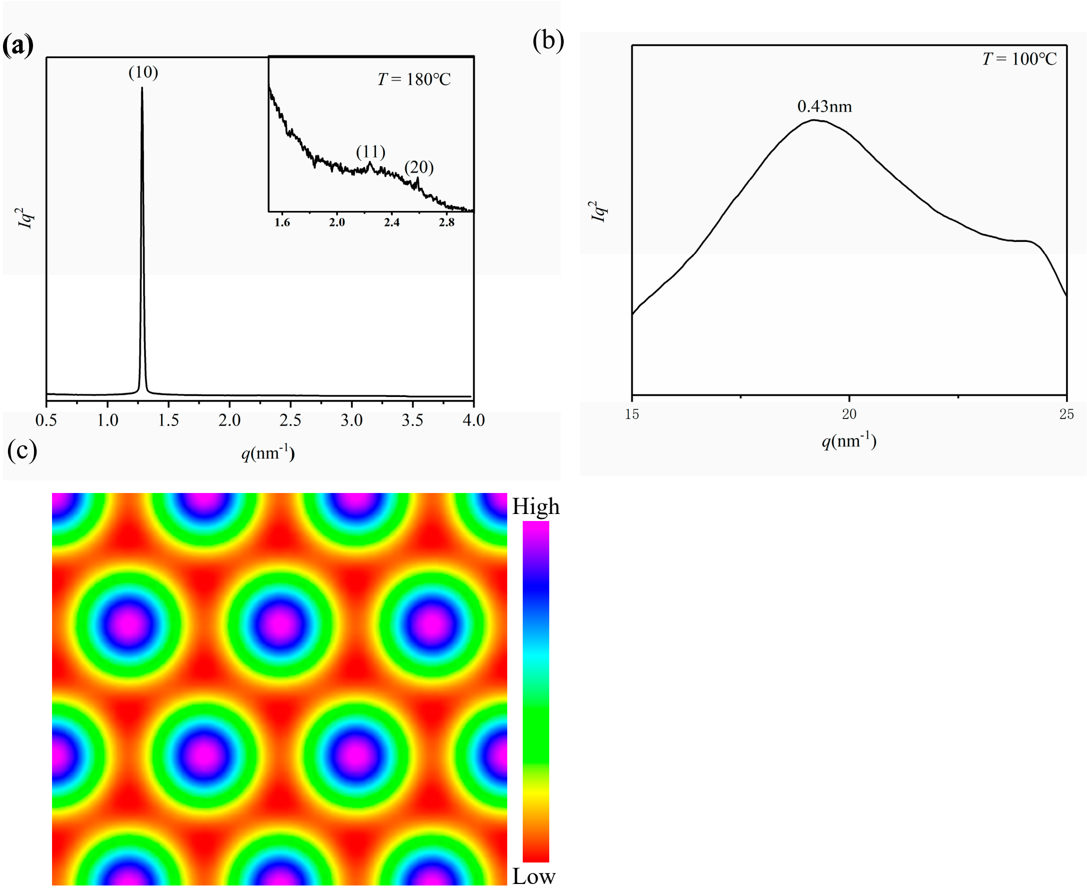

Figure S5. a) SAXS diffraction patterns of Col_{hex}/*p6mm* phase of compound **O/14** recorded at 180 °C; b) WAXS diffraction patterns the Col_{hex}/*p6mm* phase of compound **O/14** recorded at 100 °C; c) reconstructed electron density map obtained from the SAXS diffraction patterns of (a).

Table S4. Experimental and calculated *d*-spacings of the observed SAXS reflections in compound **O/16** at 180 °C.

(*hk*)	$d_{obs.}$–spacing (nm)	$d_{cal.}$–spacing (nm)	Intensity	*Phase*
(10)	4.89	4.87	100	π
(11)	2.81	2.82	0.13	0
a_{hex} = 5.64 nm				

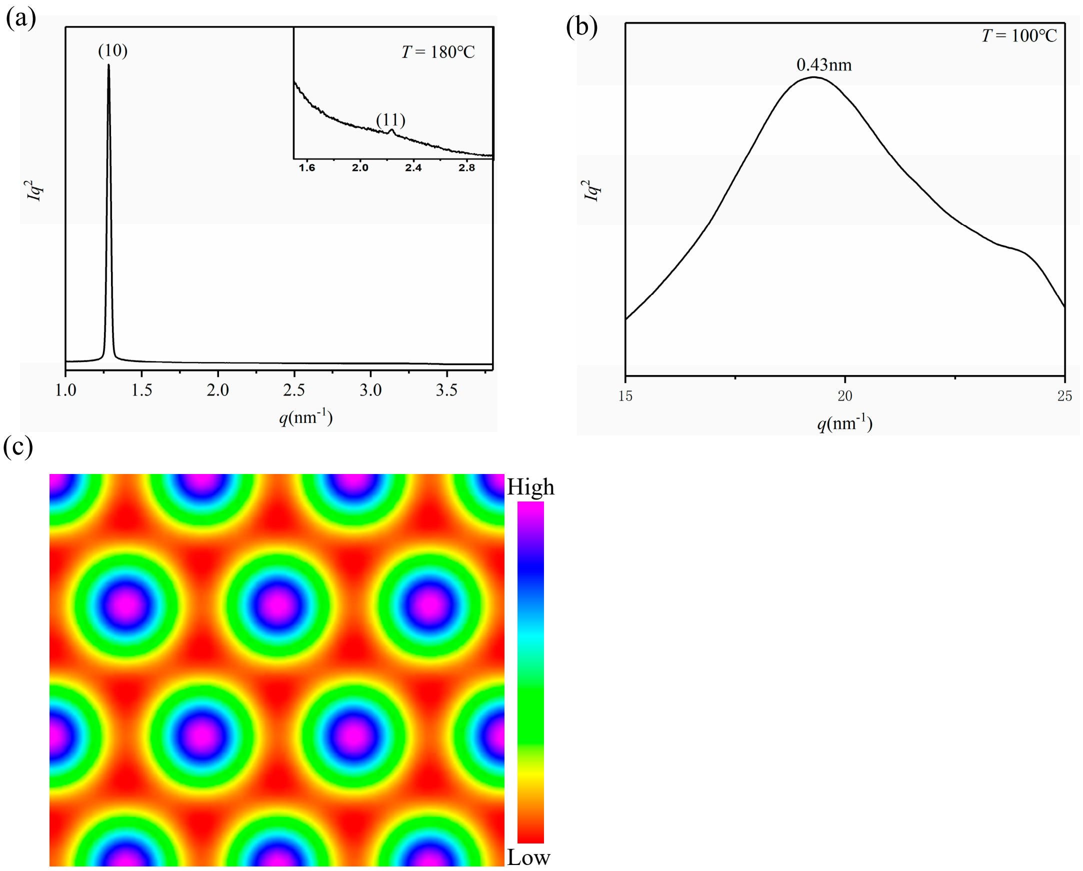

Figure S6. a) SAXS diffraction patterns of Col_{hex}/*p6mm* phase of compound **O/16** recorded at 180 °C; b) WAXS diffraction patterns the Col_{hex}/*p6mm* phase of compound **O/16** recorded at 100 °C; c) reconstructed electron density map obtained from the SAXS diffraction patterns of (a).

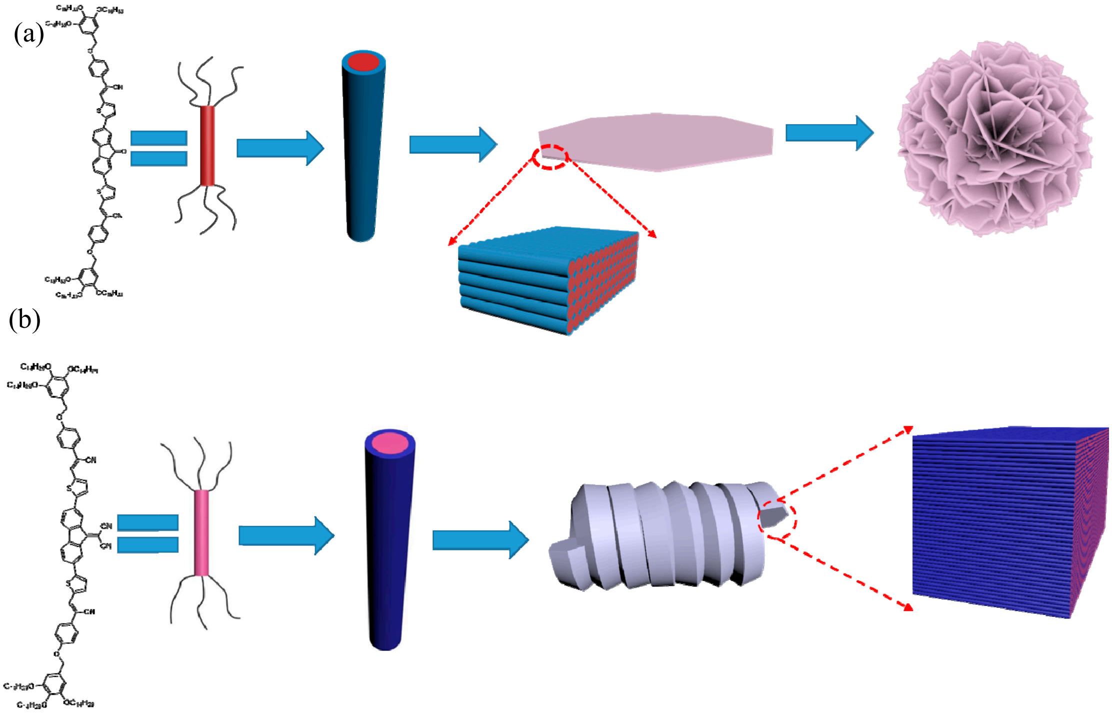

Figure S7. Schematic diagram of the possible gels formation mechanism of **O/16** and **M/14** in 1,4-dioxane solvents. a) Formation mechanism of compound **O/16** into flowerlike sphere (**O/16** in 1,4-dioxane solvents, the strong dipole-dipole interaction between gelators or gelators and solvents, as well as π-π stacking between gelators, led to the formation of a 2D hexagonal columnar structure, which gradually fused with each other to form nanosheets. the twisting and folding of the nanosheets, and finally the tangled sheets led to the formation of a spherical flowerlike structure); b) Formation mechanism of compound **M/14** into helical cylinder (Stronger π-π interactions of **M/14** molecules, led to the formation of nanopillars which further twisted and eventually became helical cylinders).

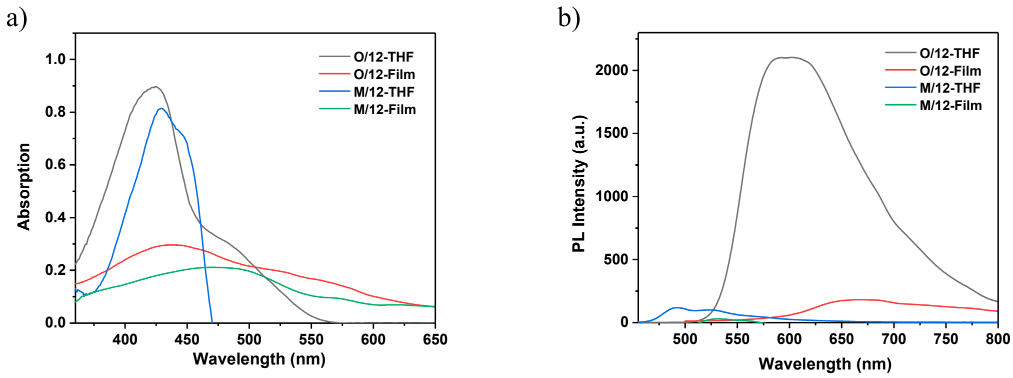

Figure S8. Normalized UV-vis absorption and PL spectra of a) **O/12** (the UV-vis absorption and excitation wavelength of **O/12** in THF is 425 nm and 602 nm, in the thin film is 437 nm and 666 nm); b) **M/12** (the UV-vis absorption and excitation wavelength of **M/12** in THF is 429 nm and 493 nm, in the thin film is 473 nm and 532 nm). (1.0×10^{-5} mol L^{-1})

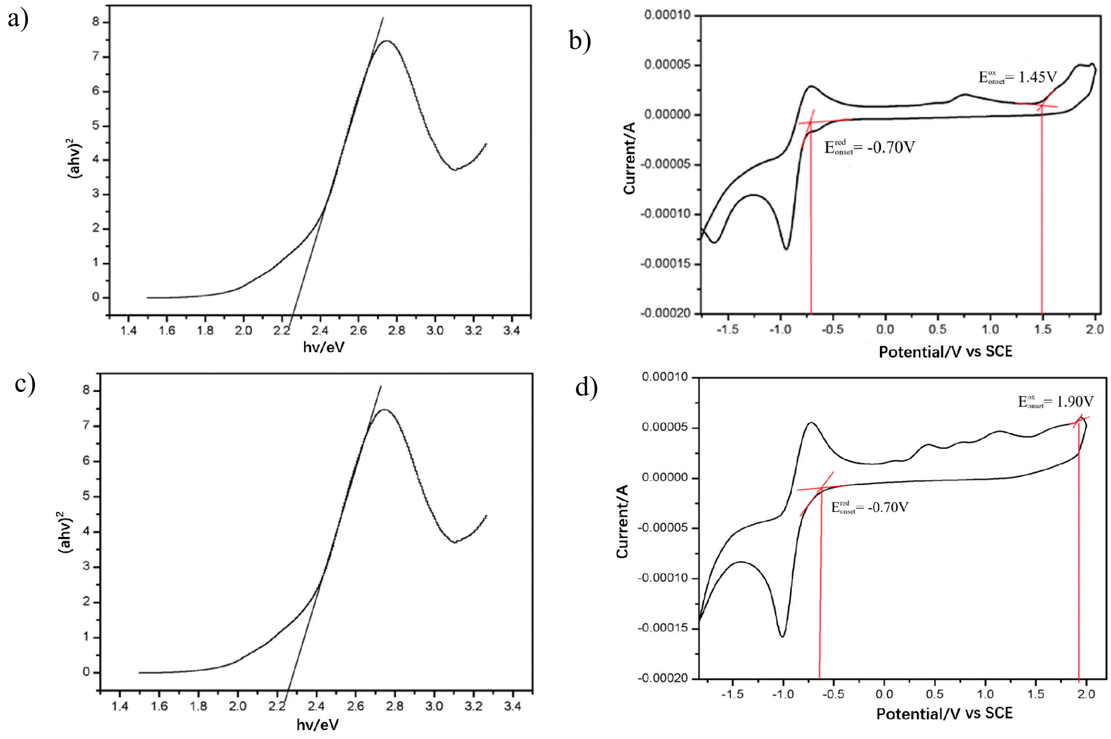

Figure S9. a) The calculation of energy band of **M/14** in film by UV-vis spectrum; b) Cyclic

voltammogram of **M/14** film on glassy carbon electrode in 0.1 mol L^{-1} Bu_4NPF_6 in acetonitrile solution with a scan rate of 50 mV s^{-1}. Reference electrode: SCE. E_{HOMO} = -(E_{ox} + 4.74 eV), E_{LUMO} = -(E_{red} + 4.74 eV), and E_g= E_{LUMO} – $E_{HOMO;}$ c) The calculation of energy band of **O/12** in film by UV-vis spectrum; d) Cyclic voltammogram of **O/12** film on glassy carbon electrode in 0.1 mol L^{-1} Bu_4NPF_6 in acetonitrile solution with a scan rate of 50 mV s^{-1}. Reference electrode: SCE. E_{HOMO} = -(E_{ox} + 4.74 eV), E_{LUMO} = -(E_{red} + 4.74 eV), and E_g= E_{LUMO} – E_{HOMO}

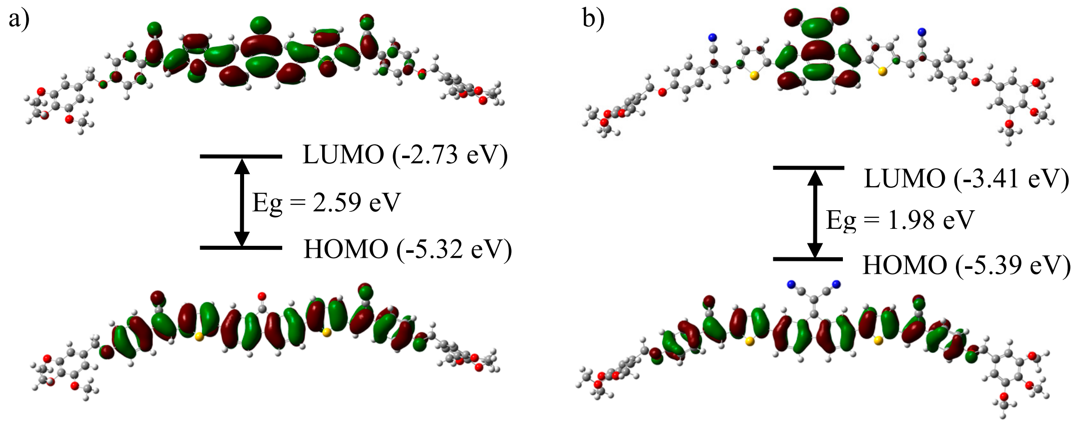

Figure S10. HOMO, LUMO orbital of (a) **O/*n***; (b) **M/*n*** calculated using the DFT/B3LYP, 6-31G (d).

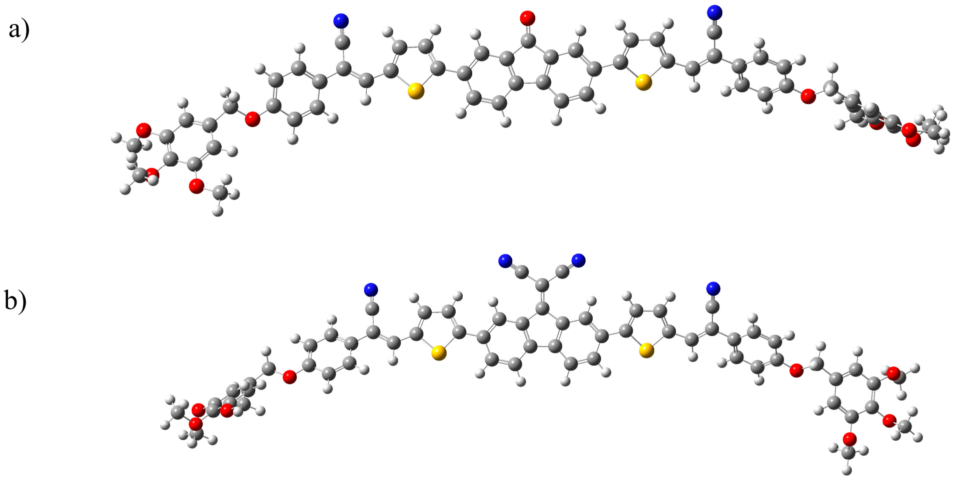

Figure S11. Geometrically optimized molecular models spatial distributions of **O/*n* and M/*n*** by TD-DFT B3LYP/6-31G (d) calculation.

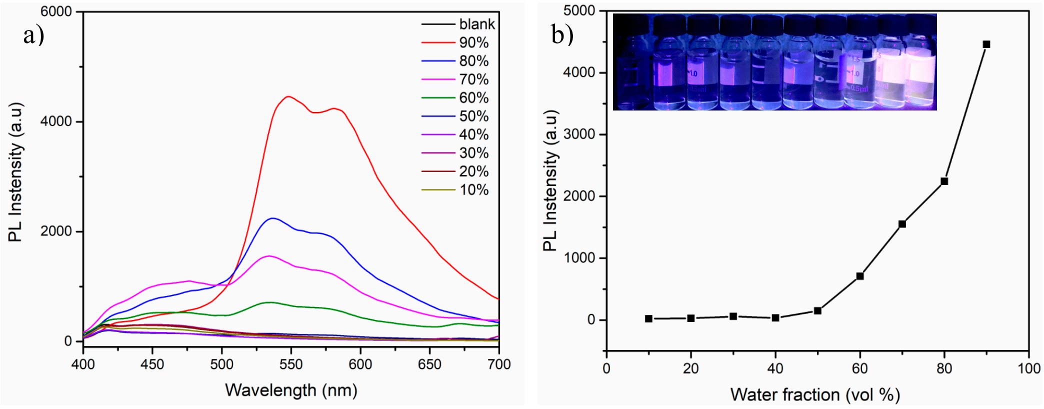

Figure S12. a) PL spectra of **10/12** in THF/water mixtures with different water fractions (*f*w); b) Plot of peak intensity of **10/12** versus water fraction in the aqueous mixtures. (The intermediate **10/12** exhibits obvious aggregation-induced enhanced emission (AIEE) properties. As shown in Figure S12a, the fluorescence intensity displayed a slow increasing tendency as the water fraction of the THF/water mixture increased from 0% to 50%. After the water fraction reached 60%, the fluorescence intensity increased significantly with the increasing water fraction up to 90%, the fluorescence intensity was 16 times higher than that in THF solvent.)

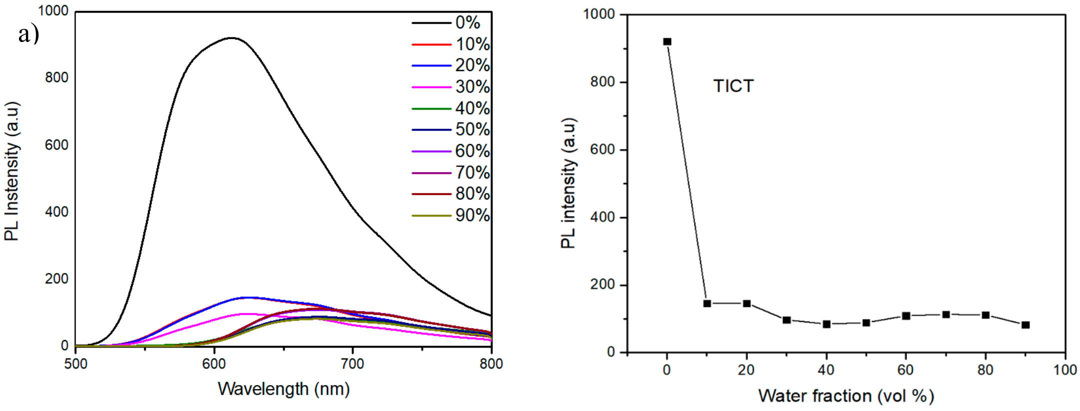

Figure S13. a) PL spectra of **O/12** in THF/water mixtures with different water fractions (*f*w); b) Plot of peak intensity of **O/12** versus water fraction in the aqueous mixtures. (With different water fractions (*f*w), which enabled fine-tuning of the solvent polarity and the extent of solute aggregation. The pure THF solution of **O/12** had an intense orange red fluorescence with an emission maximum at 609 nm, the emission became weaker as water was added with *f*w ≤ 50 vol% in the THF/water mixtures. Meanwhile, the emission maximum was bathochromically shifted to 676 nm. This is a typical twisted intramolecular charge transfer (TICT) effect arising from the increased solvent polarity. TICT, which is featured with a red-shifted emission color and a decreased emission intensity with increasing solvent polarity[S3]. When more water (*f*w > 50 vol%) was added, the **O/12** molecules formed nanoaggregates. Although a hydrophobic environment was created inside the nanoaggregates and the TICT effect should be alleviated, the fluorescence did not recover.

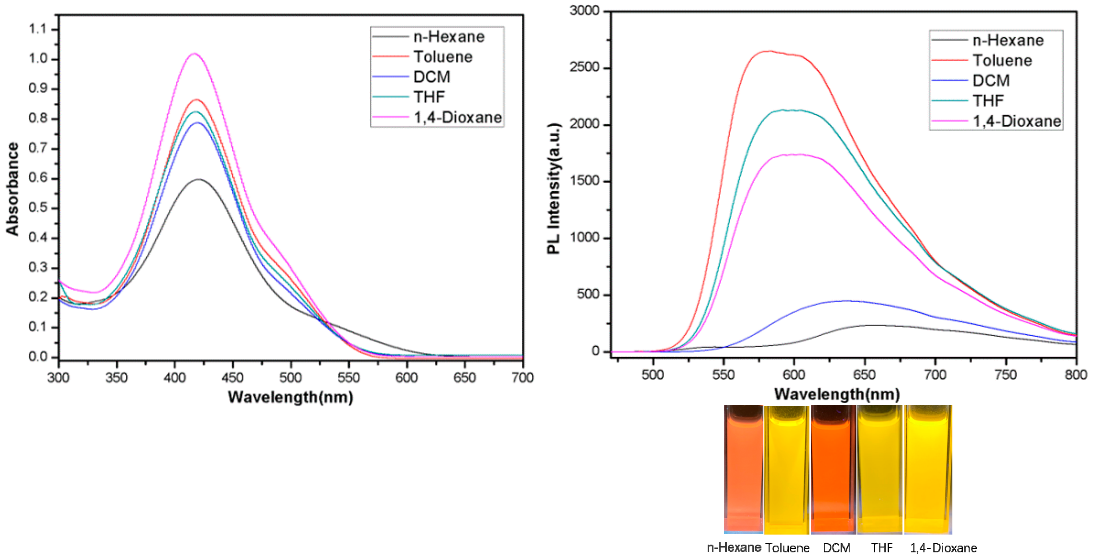

Figure S14. a) UV-vis absorption spectra of **O/12** in different organic solvents (1.0×10^{-5} mol L^{-1}); b) PL spectra of **O/12** in different organic solvents (1.0×10^{-5} mol L^{-1}) excited at maximum absorption peaks.

2 Materials, synthesis and analytical data

2.1 Materials and synthesis

Reactions requiring an inert gas atmosphere were conducted under argon and the glassware was oven-dried (140 ℃). Tetrahydrofuran (THF) was distilled from sodium prior to use. All reagents were purchased from commercial sources and used as received. ^{1}H NMR and ^{13}C NMR spectra were recorded on a Bruker-DRX-400 spectrometer. Thin-layer chromatography was performed on aluminum plates precoated with 5735 silica gel 60 PF254 (Merck). Column chromatography was performed on Merck silica gel 60 (230-400 mesh).

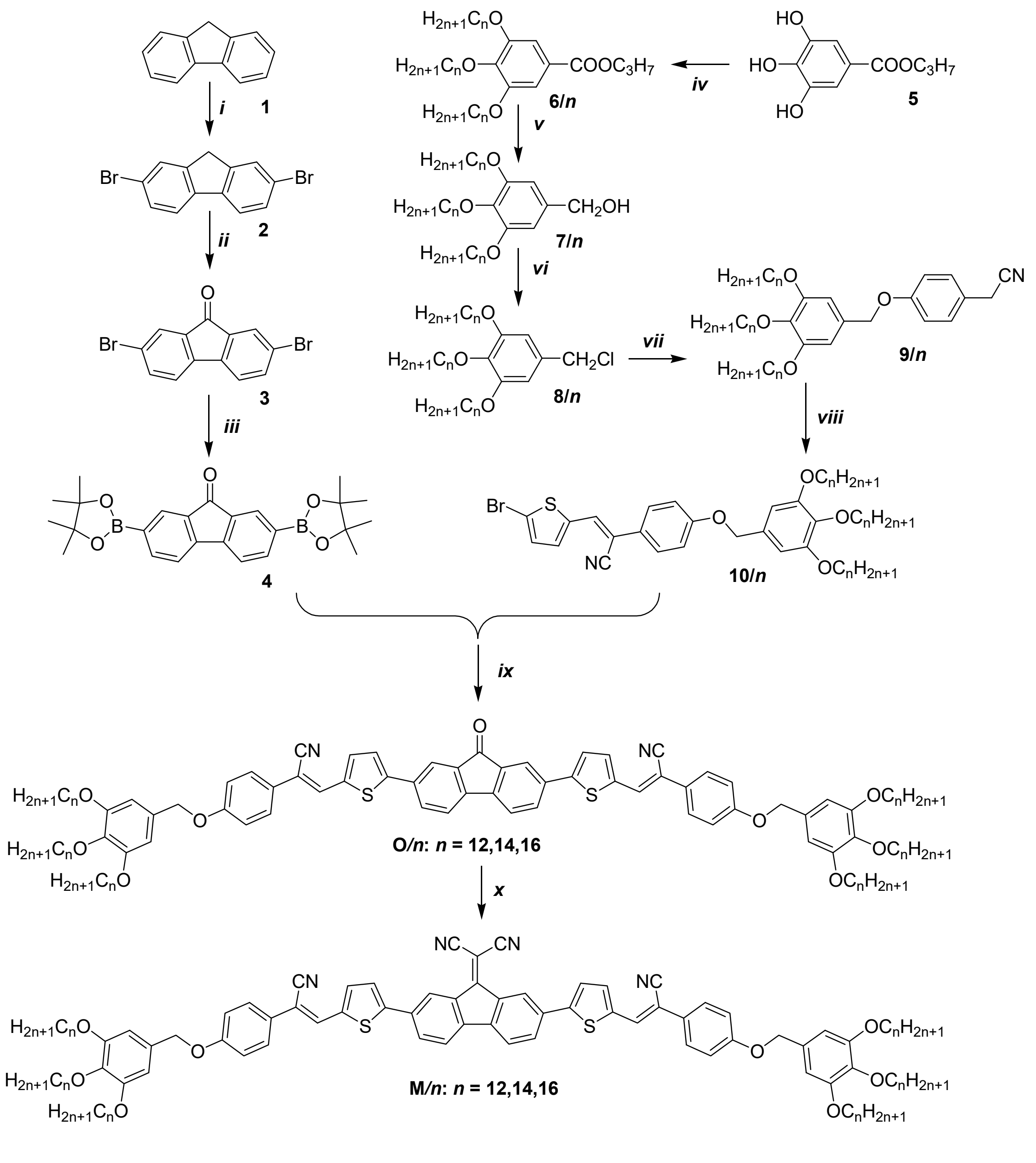

Scheme S1. Synthesis of compounds **O/*n*** and **M/*n***: Reagents and conditions: *i*) Br_2, $FeCl_3$, $CHCl_3$, 0 °C, 4 h; *ii*) CH_3COOH, CrO_3, RT, 24 h; *iii*) bis(pinacolato)diboron, CH_3COOK, $PdCl_2$(dppf), 1,4-dioxane, N_2, 100 °C, 12 h; *iv*) RBr, K_2CO_3, DMF, 12 h; *v*) $LiAlH_4$, THF, r.t., 3h; *vi*) $SoCl_2$, THF, 0 °C, 1 h; *vii*) 4-Hydroxybenzeneacetonitrile, K_2CO_3, CH_3CN, 75 °C, 12 h; *viii*) 5-Bromothiophene-2-Carbaldehyde, CH_3ONa, Ethanol, 80 °C, 12 h; *ix*) $Pd(PPh_3)_4$, K_2CO_3, THF, H_2O, N_2, 75 °C, 24 h; *x*) $CH_2(CN)_2$, pyridine, 80 °C, 2 h.

Synthesis of 2,7-dibromofluorene **2**[S4]

Fluorene (600 mg, 3.61 mmol) was dissolved in chloroform (10 mL) and $FeCl_3$ (10 mg, 0.06 mmol) was added. The solution was cooled to 0 °C, and add bromine (0.33 mL, 6.34 mmol) dropwise to the mixture while stirring. The mixture was stirred for an additional three hours. After the reaction was complete (TLC), saturated $Na_2S_2O_3$ solution (20 mL) was slowly added and stirring for 30 minutes. Then the mixture was extracted with

dichloromethane (3 × 50 mL). The combined organic layers were dried over anhydrous Na_2SO_4, filtered and the solvent was evaporated in *vacuo*. The crude product was purified by column chromatography (Petroleum ether) to produce compound **2** as white solid. Yield: 1.11 g (95.6%). ^{1}H-NMR (300 MHz, $CDCl_3$), δ (ppm): 7.64-7.62 (d, J = 7.2 Hz, 2H, Ph**H**), 7.58-7.52 (t, J = 9.0 Hz, 2H, Ph**H**), 7.49-7.46 (d, J = 8.1 Hz, 2H, Ph**H**), 3.84-3.78 (d, J = 15.6 Hz, 2H, fluorene-9**H**).

Synthesis of 2,7-dibromo-9-fluorenone **3**[S5]

A mixture of 2,7-dibromofluorene **2** (1.11 g, 3.45 mmol) and CrO_3 (4.00 g, 40.0 mmol) suspended in 50 mL acetic acid, and stirring at room temperature for 12 h. After the reaction was complete (TLC), the resulting yellow precipitate was collected by suction filtration, washed with water thoroughly, and dried under vacuum to provide the product as yellow solid. The crude product was purified by column chromatography (Petroleum ether) to produce compound **3** as white solid. Yield: 466.44 mg (40.2%). ^{1}H-NMR (400 MHz, $CDCl_3$), δ (ppm): 7.78 (s, 2H, Ph**H**), 7.64-7.62 (d, J = 8.0 Hz, 2H, Ph**H**), 7.40-7.39 (d, J = 7.6 Hz, 2H, Ph**H**).

Synthesis of 2,7-bis(4,4,5,5-tetramethyl-1,3,2-dioxaborolan-2-yl)-9H-fluoren-9-one **4**[S6]

To a previously degassed 1,4-dioxane (25 mL) solution of 2,7-dibromo-9-fluorenone (466.44 mg, 1.38 mmol), bis(pinacolato)diboron (1.4 g, 5.52 mmol), $PdCl_2$(dppf) (20 mg) and KOAc (677.16 mg, 6.9 mmol) were added and the mixture was refluxed at 100 °C overnight. After the reaction was complete (TLC), the mixture was cooled to RT and the dioxane was removed under vacuum, and then then the mixture was extracted with dichloromethane (3 × 100 mL). The combined organic phase was dried over anhydrous $MgSO_4$. After the solvent was evaporated in *vacuo*, the crude product was purified by column chromatography (petroleum ether : ethyl acetate = 15 : 1) to get produce pure compounds **4** as yellow solid. Yield: 180.1 mg (30.2%). ^{1}H-NMR (400 MHz, $CDCl_3$), δ (ppm): 8.13 (s, 2H, Ph**H**), 7.96-7.94 (d, J = 7.4 Hz, 2H, Ar**H**), 7.57-7.55 (d, J = 7.4 Hz, 2H, Ph**H**), 1.35 (s, 24H, 8 C-C**H**$_3$).

General procedure for the synthesis of compounds 6/*n*, 7/*n* and **8/*n***

Compounds **6/*n***, **7/*n*** and **8/*n*** were synthesized according to literature procedures in ref [S7].

General procedure for the synthesis of 9/*n*[S8]

To a mixture of K_2CO_3 (8.2 mmol, 1.31 g), 4-hydroxybenzyl cyanide (1.64 mmol, 218.36 mg), dry CH_3CN (40 mL), the appropriate **8/*n*** (0.82 mmol) was added. The mixture was heated to 80 °C and refluxed for 12 h. After the reaction was complete (TLC), the mixture was cooled to RT, H_2O (15 mL) was added to dissolve the precipitate and then the reaction mixture was extracted with ethyl acetate (3 × 60 mL). The combined organic phase was dried by anhydrous Na_2SO_4. The solvent was evaporated under reduced pressure and then purified by column chromatography (petroleum ether : ethyl acetate =

30 : 1) to afford white solid compounds.

9/12: Yield: 86%. ^{1}H-NMR (400 MHz, $CDCl_3$), *δ* (ppm): 7.25-7.23 (d, *J* = 8.56 Hz, 2H, Ph**H**), 6.98-6.96 (d, *J* = 7.52 Hz, 2H, Ph**H**), 6.61 (s, 2H, Ph**H**), 4.94 (s, 2H, PhO-C**H**$_2$Ph), 3.98-3.93 (m, 6H, 3OC**H**$_2$), 3.69 (s, 2H, C**H**$_2$CN), 1.81-1.72 (m, 6H, OCH_2C**H**$_2$), 1.46 (m, 12H, 3OCH_2CH_2C**H**$_2$), 1.26 (m, 48H, 24C**H**$_2$), 0.89-0.86 (t, *J* = 6.26 Hz, 9H, 3C**H**$_3$).
9/14: Yield: 87%. ^{1}H-NMR ($CDCl_3$, 400 MHz): *δ* = 7.25-7.23 (d, *J* = 8.4 Hz, 2 H, 2 Ph**H**), 6.98-6.96 (d, *J* = 7.6 Hz, 2 H, 2 Ph**H**), 6.61 (s, 2 H, 2 Ph**H**), 4.94 (s, 2 H, PhO-C**H**$_2$Ph), 3.99-3.93 (m, 6 H, 3 OC**H**$_2$), 3.70 (s, 2 H, C**H**$_2$CN), 1.81-1.72 (m, 6 H, 3 OCH_2C**H**$_2$), 1.46 (s, 6 H, 3 OCH_2CH_2C**H**$_2$), 1.26 (s, 60 H, 30 C**H**$_2$), 0.89-0.86 (t, *J* = 6.2 Hz, 9 H, 3 C**H**$_3$).
9/16: Yield: 84%. ^{1}H-NMR ($CDCl_3$, 400 MHz): δ (ppm): 7.24 (d, J = 8.4 Hz, 2H, 2 Ph**H**), 6.98-6.96 (m, 2H, 2 Ph**H**), 6.60 (s, 2H, 2 Ph**H**), 4.94 (s, 2H, PhO-C**H**$_2$Ph), 3.99-3.93 (m, 6H, 3 OC**H**$_2$), 3.69 (s, 2H, C**H**$_2$CN), 1.82-1.70 (m, 6H, 3 OCH_2C**H**$_2$), 1.46-1.42 (m, 6H, 3 OCH_2CH_2C**H**$_2$), 1.38-1.21 (m, 72H, 36 C**H**$_2$), 0.88 (t, J = 6.8 Hz, 9H, 3 C**H**$_3$).

General procedure for the synthesis of 10/*n*

A solution of the 5-bromothiophene-2-carbaldehyde (4 mmol, 764.0 mg) and **9/*n*** (4 mmol) in absolute EtOH (20 mL) was treated with NaOMe (4 mmol, 272.2 mg) portionwise, stirred at room temperature for 1 h. After the reaction was complete (TLC), the mixture was cooled to 0 °C, and filtered. The precipitate was washed with EtOH.

10/12: Yield: 82%. ^{1}H-NMR ($CDCl_3$, 400 MHz): *δ* = 7.57-7.54 (d, *J* = 8.7 Hz, 2 H, 2 Ph**H**), 7.43 (s, 1 H, 1 C**H**=R), 7.30-7.29 (d, *J* = 4.1 Hz, 1 H, 1 Ph**H**), 7.10-7.09 (d, *J* = 4.0 Hz, 1 H, 1 Ph**H**), 7.03-7.01 (d, *J* = 4.3 Hz, 2 H, 2 Ph**H**), 6.61 (s, 2 H, 2 Ph**H**), 4.98 (s, 2 H, Ph-C**H**$_2$), 3.99-3.94 (m, 6 H, 3 PhOC**H**$_2$), 1.83-1.71 (m, 6 H, 3 OCH_2C**H**$_2$), 1.46-1.42 (m, 6 H, 3 OCH_2CH_2C**H**$_2$), 1.26 (s, 48 H, 24 C**H**$_2$), 0.89-0.86 (t, *J* = 6.7 Hz, 9 H, 3 C**H**$_3$).
10/14: Yield: 87%. ^{1}H-NMR ($CDCl_3$, 400 MHz): *δ* = 7.57-7.55 (d, *J* = 8.8 Hz, 2 H, 2 Ph**H**), 7.42 (s, 1 H, 1 C**H**=R), 7.30-7.29 (d, *J* = 4.0 Hz, 1 H, 1 Ph**H**), 7.10-7.09 (d, *J* = 4.0 Hz, 1 H, 1 Ph**H**), 7.03-7.01 (d, *J* = 4.4 Hz, 2 H, 2 Ph**H**), 6.61 (s, 2 H, 2 Ph**H**), 4.98 (s, 2 H, Ph-C**H**$_2$), 3.99-3.93 (m, 6 H, 3 PhOC**H**$_2$), 1.83-1.71 (m, 6 H, 3 OCH_2C**H**$_2$), 1.46-1.42 (m, 6 H, 3 OCH_2CH_2C**H**$_2$), 1.26 (s, 60 H, 30 C**H**$_2$), 0.89-0.86 (t, *J* = 6.6 Hz, 9 H, 3 C**H**$_3$).
10/16: Yield: 88%. ^{1}H-NMR ($CDCl_3$, 400 MHz): *δ* = 7.57-7.55 (d, *J* = 8.8 Hz, 2 H, 2 Ph**H**), 7.42 (s, 1 H, 1 C**H**=R), 7.30-7.29 (d, *J* = 4.0 Hz, 1 H, 1 Ph**H**), 7.10-7.09 (d, *J* = 4.0 Hz, 1 H, 1 Ph**H**), 7.03-7.01 (d, *J* = 4.4 Hz, 2 H, 2 Ph**H**), 6.61 (s, 2 H, 2 Ph**H**), 4.98 (s, 2 H, Ph-C**H**$_2$), 3.99-3.93 (m, 6 H, 3 PhOC**H**$_2$), 1.83-1.71 (m, 6 H, 3 OCH_2C**H**$_2$), 1.46-1.42 (m, 6 H, 3 OCH_2CH_2C**H**$_2$), 1.26 (s, 72 H, 36 C**H**$_2$), 0.89-0.86 (t, *J* = 6.6 Hz, 9 H, 3 C**H**$_3$).

General procedure for the synthesis of the compounds O/*n*

A mixture of the compound **4** (45 mg, 0.1 mmol), compound **10/*n*** (0.25 mmol), K_2CO_3 (72 mg, 0.4 mmol), $Pd(PPh_3)_4$ (5 mg), THF (15 mL) and H_2O (5 mL) was refluxed at 78 °C for 24 h under an argon atmosphere. After the reaction was complete (TLC), the mixture was cooled to RT, and then the reaction mixture was extracted with dichloromethane (3 × 50 mL). The combined organic layer was dried with anhydrous Na_2SO_4, and the solvent was

evaporated in *vacuo*. The residue was purified by column chromatography (petroleum ether/ dichloromethane = 1 : 2) to produce compounds **O/*n*** as red solid.

O/12: Yield: 79.9 mg, 41.7%. ^{1}H-NMR (400 MHz, $CDCl_3$), δ (ppm): 7.76 (s, 2H, Ph**H**), 7.67-7.65 (d, *J* = 7.84 Hz, 2H, Ph**H**), 7.52-7.50 (d, *J* = 8.36 Hz, 4H, Ph**H**), 7.45-7.43 (m, 4H, 2Ph**H,** 2 Thiophene**H**), 7.38 (s, 2H, 2C**H**), 7.31-7.30 (d, *J* = 2.88 Hz, 2H, Thiophene**H**), 6.97-6.96 (d, *J* = 8.32 Hz, 4H, Ph**H**), 6.60 (s, 4H, Ph**H**), 4.92 (s, 4H, 2ArOC**H**$_2$Ar), 3.98-3.93 (m, 12H, 6OC**H**$_2$), 1.81-1.73 (m, 12H, 6OCH$_2$C**H**$_2$), 1.46 (m, 12H, 6OCH$_2$CH$_2$C**H**$_2$), 1.26 (m, 96H, 6OCH$_2$CH$_2$CH$_2$(C**H**$_2$)$_8$), 0.89-0.86 (t, *J* = 6.04Hz, 18H, 6C**H**$_3$). ^{13}C-NMR (100 MHz, $CDCl_3$): δ = 192.65, 159.51, 153.38, 146.38, 143.23, 138.09, 137.92, 135.01, 134.35, 133.38, 131.84, 131.59, 131.31, 126.97, 126.41, 124.15, 121.24, 121.09, 118.20, 115.37, 107.65, 106.07, 73.46, 70.49, 69.18, 31.95, 30.39-14.13(Alkyl chain). Elemental analysis calcd (%) for $C_{125}H_{178}N_2O_9S_2$ (1915.3); C, 78.32; H, 9.36; N, 1.46; Found: C, 78.27; H, 9.29; N, 1.43.

O/14: Yield: 90.7 mg, 43.5%. ^{1}H-NMR (400 MHz, $CDCl_3$), δ (ppm): 7.72 (s, 2H, Ph**H**), 7.64-7.62 (d, *J* = 7.8 Hz, 2H, Ph**H**), 7.51-7.49 (d, *J* = 8.72 Hz, 4H, Ph**H**), 7.43-7.42 (d, *J* = 3.96 Hz, 2H, Thiophene**H**), 7.41-7.38 (m, 2H, Ph**H)**, 7.35 (s, 2H, 2C**H**), 7.28-7.27 (d, *J* = 3.64 Hz, 2H, Thiophene**H**), 6.97-6.94 (d, *J* = 8.76 Hz, 4H, Ph**H**), 6.59 (s, 4H, Ph**H**), 4.90 (s, 4H, 2ArOC**H**$_2$Ar), 4.00-3.93 (m, 12H, 6OC**H**$_2$), 1.82-1.71 (m, 12H, 6OCH$_2$C**H**$_2$), 1.48-1.43 (m, 12H, 6OCH$_2$CH$_2$C**H**$_2$), 1.26 (m, 120H, 6OCH$_2$CH$_2$CH$_2$(C**H**$_2$)$_{10}$), 0.89-0.86 (t, *J* = 6.78 Hz, 18H, 6C**H**$_3$). ^{13}C-NMR (100 MHz, $CDCl_3$): δ = 192.46, 159.49, 153.38, 146.35, 143.19, 138.08, 137.89, 134.97, 134.29, 133.37, 131.77, 131.53, 131.31, 126.94, 126.39, 124.12, 121.15, 121.05, 118.18, 115.35, 107.60, 106.05, 73.45, 70.47, 69.18, 31.95, 30.39-14.13(Alkyl chain). Elemental analysis calcd (%) for $C_{137}H_{202}N_2O_9S_2$ (2083.49); C, 78.91; H, 9.76; N, 1.34; Found: C, 78.89; H, 9.83; N, 1.37.

O/16: Yield: 99.16 mg, 44.01%. ^{1}H-NMR (400 MHz, $CDCl_3$), δ (ppm): 7.82 (s, 2H, Ph**H**), 7.71-7.69 (d, *J* = 8.04 Hz, 2H, Ph**H**), 7.55-7.53 (d, *J* = 8.6 Hz, 4H, Ph**H**), 7.48-7.47 (d, *J* = 3.64 Hz, 2H, Thiophene**H**), 7.46-7.45 (m, 2H, Ph**H)**, 7.42 (s, 2H, 2C**H**), 7.34-7.33 (d, *J* = 3.92 Hz, 2H, Thiophene**H**), 6.99-6.97 (d, *J* = 8.6 Hz, 4H, Ph**H**), 6.61 (s, 4H, Ph**H**), 4.94 (s, 4H, 2ArOC**H**$_2$Ar), 4.02-3.93 (m, 12H, 6OC**H**$_2$), 1.83-1.71 (m, 12H, 6OCH$_2$C**H**$_2$), 1.48-1.43 (m, 12H, 6OCH$_2$CH$_2$C**H**$_2$), 1.25 (m, 144H, 6OCH$_2$CH$_2$CH$_2$(C**H**$_2$)$_{12}$), 0.89-0.86 (t, *J* = 6.8 Hz, 18H, 6C**H**$_3$). ^{13}C-NMR (100 MHz, $CDCl_3$): δ = 192.58, 159.53, 153.38, 146.42, 143.30, 138.10, 137.96, 135.07, 134.44, 133.38, 131.94, 131.67, 131.31, 127.00, 126.46, 124.19, 121.37, 121.12, 118.21, 115.40, 107.75, 106.09, 76.70, 73.46, 70.52, 69.18, 31.94, 30.38-14.13(Alkyl chain). Elemental analysis calcd (%) for $C_{149}H_{226}N_2O_9S_2$ (2251.67); C, 79.41; H, 10.11; N, 1.24; Found: C, 79.38; H, 10.18; N, 1.20.

General procedure for the synthesis of the compounds M/*n*.

Malononitrile (8.58 mg, 0.13 mmol) and compound **O/*n*** (30 mg, 0.014 mmol) were dissolved in dry pyridine (10 mL) and the solution was stirred at 20 °C for 1 h to obtain a brown suspension. Pyridine (10 mL) was added and the mixture was stirred for an additional 5 h, followed by heating to 80 °C for 1 h. Ethanol (20 mL) was added to the solution and the mixture was cooled to 20 °C. A green solid of **M/*n*** was obtained after suction filtration and washed by acetonitrile.

M/12: Yield: 22 mg, 73.6%. ^{1}H-NMR (400 MHz, $CDCl_3$), δ (ppm): 8.32 (s, 2H, Ph**H**), 7.65-7.63 (d, *J* = 8.12 Hz, 2H, Ph**H**), 7.48-7.46 (d, *J* = 7.96 Hz, 2H, Ph**H**), 7.44-7.41 (d, *J* = 7.96 Hz, 4H, Ph**H**), 7.40-7.39 (d, *J* = 4.32 Hz, 2H, Thiophene**H**), 7.26 (s, 2H, C**H**), 7.22-7.21 (d, *J* = 3.76 Hz, 2H, Thiophene**H**), 6.91-6.89 (d, *J* = 8.72 Hz, 4H, Ph**H**), 6.58 (s, 4H, Ph**H**), 4.85 (s, 4H, 2ArOC**H**$_2$Ar), 3.97-3.94 (m, 12H, 6OC**H**$_2$), 1.80-1.73 (m, 12H, 6OCH$_2$C**H**$_2$), 1.46 (m, 12H, 6OCH$_2$CH$_2$C**H**$_2$), 1.27 (m, 96H, 6OCH$_2$CH$_2$CH$_2$(C**H**$_2$)$_8$), 0.90-0.87 (t, *J* = 6.62 Hz, 18H, 6C**H**$_3$). ^{13}C-NMR (100 MHz, $CDCl_3$): δ = 159.49, 153.37,145.09, 140.67, 138.20, 138.09, 134.74, 134.11, 132.87, 131.31, 130.53, 126.79, 125.97, 124.45, 122.88, 121.41, 117.93, 115.27, 112.95, 107.83, 105.96, 73.45, 70.29, 69.20, 31.95,30.43-14.11 (Alkyl chain). Elemental analysis calcd (%) for $C_{128}H_{178}N_4O_8S_2$ (1963.31); C, 78.24; H, 9.13; N, 2.85; Found: C, 78.32; H, 9.07; N, 2.91.

M/14: Yield: 20 mg, 66.9%. ^{1}H-NMR (400 MHz, $CDCl_3$), δ (ppm): 8.45 (s, 2H, Ph**H**), 7.71-7.69 (d, *J* = 8.12 Hz, 2H, Ph**H**), 7.53-7.51 (d, *J* = 7.96 Hz, 2H, Ph**H**), 7.49-7.47 (d, *J* = 7.96 Hz, 4H, Ph**H**), 7.46-7.45 (d, *J* = 4.32 Hz, 2H, Thiophene**H**), 7.34 (s, 2H, C**H**), 7.29-7.28 (d, *J* = 3.76 Hz, 2H, Thiophene**H**), 6.94-6.92 (d, *J* = 8.72 Hz, 4H, Ph**H**), 6.59 (s, 4H, Ph**H**), 4.88 (s, 4H, 2ArOC**H**$_2$Ar), 3.97-3.92 (m, 12H, 6OC**H**$_2$), 1.82-1.71 (m, 12H, 6OCH$_2$C**H**$_2$), 1.46 (m, 12H, 6OCH$_2$CH$_2$C**H**$_2$), 1.26 (m, 120H, 6OCH$_2$CH$_2$CH$_2$(C**H**$_2$)$_{10}$), 0.89-0.86 (t, *J* = 6.62 Hz, 18H, 6C**H**$_3$). ^{13}C-NMR (100 MHz, $CDCl_3$): δ = 159.55, 153.37, 145.40, 138.30, 138.03, 134.99, 134.42, 133.14, 131.29, 131.10, 126.95, 126.20, 124.59, 123.43, 121.52, 118.10, 115.36, 108.02, 106.00, 73.46, 70.45, 69.16, 31.96, 30.40-14.14(Alkyl chain). Elemental analysis calcd (%) for $C_{140}H_{202}N_4O_8S_2$ (2131.5); C, 78.82; H, 9.54; N, 2.63; Found: C, 78.78; H, 9.58; N, 2.60.

M/16: Yield: 17 mg, 56.9%. ^{1}H-NMR (400 MHz, $CDCl_3$), δ (ppm): 8.52 (s, 2H, Ph**H**), 7.74-7.72 (d, *J* = 8.12 Hz, 2H, Ph**H**), 7.55-7.52 (d, *J* = 7.96 Hz, 2H, Ph**H**), 7.50-7.38 (d, *J* = 7.96 Hz, 4H, Ph**H**), 7.50-7.38 (d, *J* = 4.32 Hz, 2H, Thiophene**H**), 7.38 (s, 2H, C**H**), 7.33 (d, *J* = 3.76 Hz, 2H, Thiophene**H**), 6.98-6.96 (d, *J* = 8.72 Hz, 4H, Ph**H**), 6.60 (s, 4H, Ph**H**), 4.92 (s, 4H, 2ArOC**H**$_2$Ar), 3.98-3.95 (m, 12H, 6OC**H**$_2$), 1.82-1.73 (m, 12H, 6OCH$_2$C**H**$_2$), 1.47 (m, 12H, 6OCH$_2$CH$_2$C**H**$_2$), 1.26 (m, 144H, 6OCH$_2$CH$_2$CH$_2$(C**H**$_2$)$_{12}$), 0.89-0.86 (t, *J* = 6.62 Hz, 18H, 6C**H**$_3$). ^{13}C-NMR (100 MHz, $CDCl_3$): δ = 159.58, 153.39, 145.35, 140.92, 138.31, 134.97, 133.05, 131.62, 131.30, 126.94, 126.21, 124.57, 121.48, 118.06, 115.39, 113.04, 108.06, 106.09, 73.46, 70.45, 69.21, 31.96, 30.40-14.10 (Alkyl chain). Elemental analysis calcd (%) for $C_{152}H_{226}N_4O_8S_2$ (2299.68); C, 79.32; H, 9.90; N, 2.43; Found: C, 79.31; H, 9.99; N, 2.37.

2.2 ^{1}H and ^{13}C NMR spectra for representative compounds

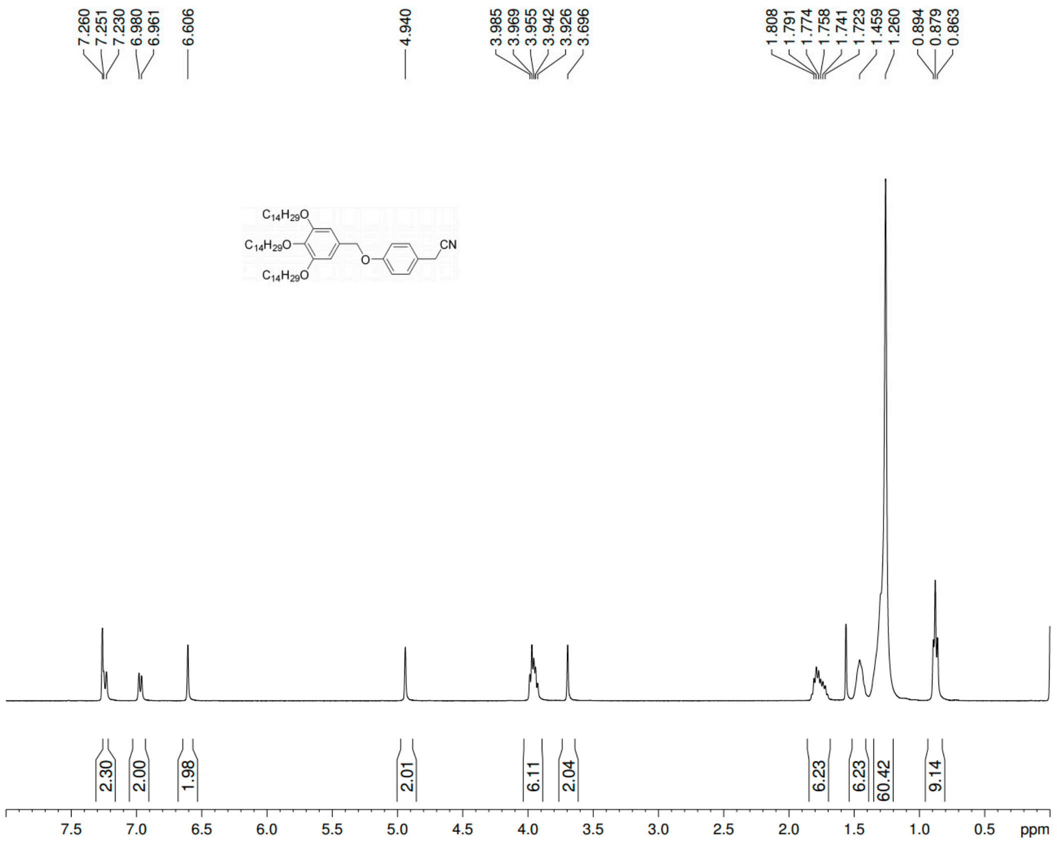

Figure S15. ^{1}H NMR ($CDCl_3$, 400 MHz ppm) spectra of **9/14**.

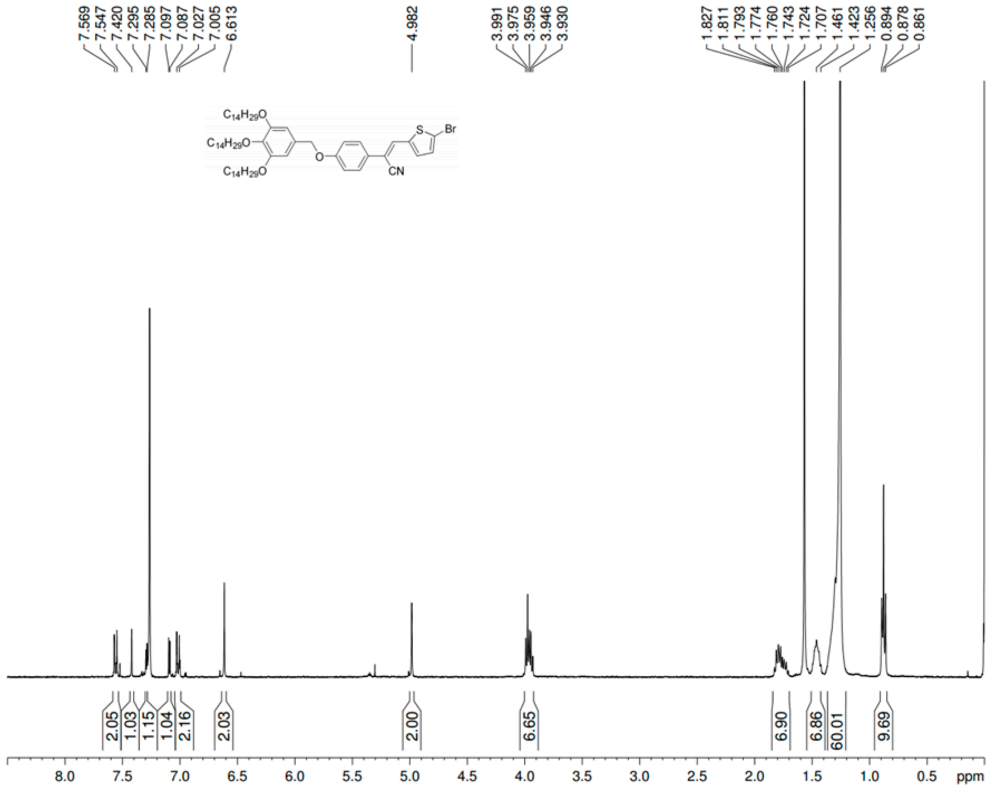

Figure S16. ^{1}H NMR ($CDCl_3$, 400 MHz ppm) spectra of **10/14**.

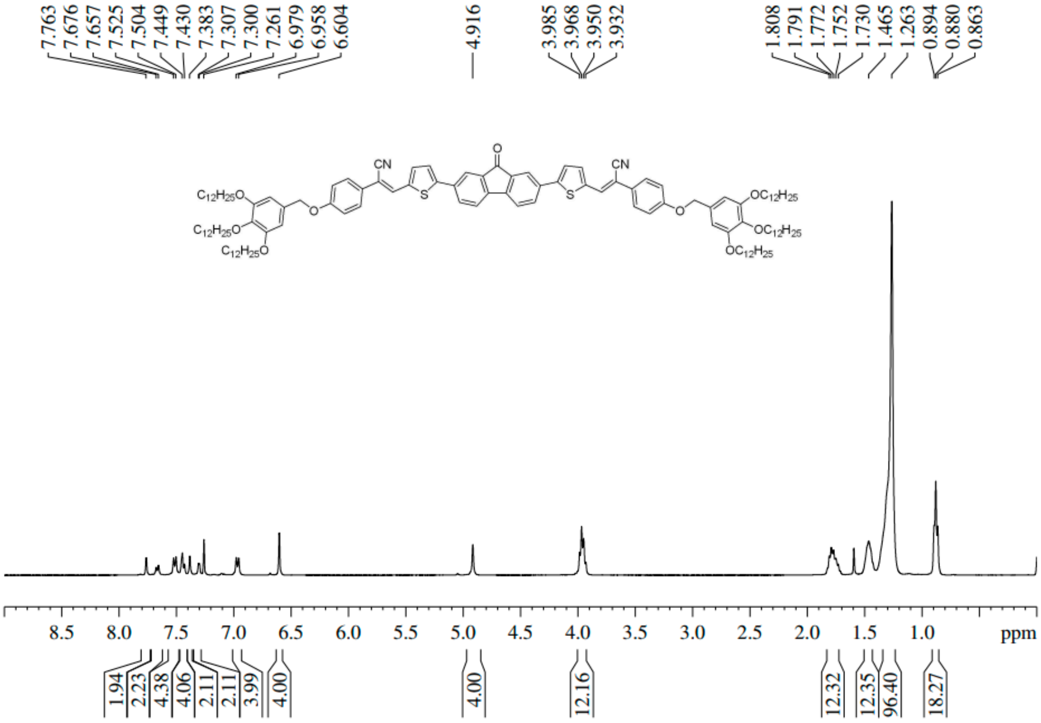

Figure S17. ^{1}H NMR ($CDCl_3$, 400 MHz ppm) spectra of **O/12**.

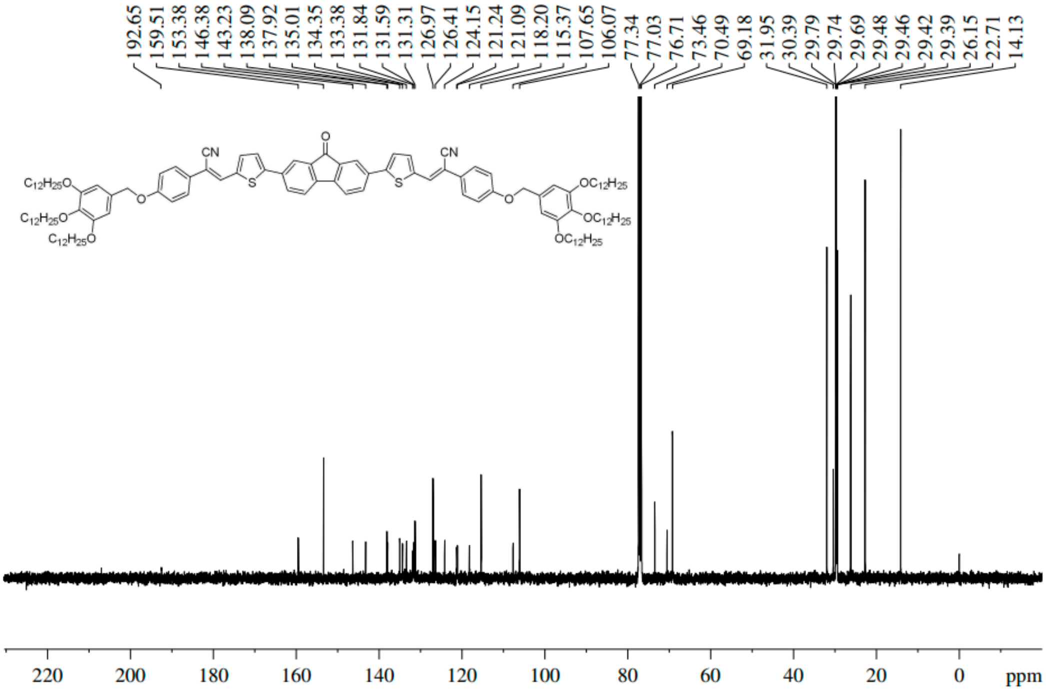

Figure S18. ^{13}C NMR ($CDCl_3$, 100 MHz ppm) spectra of **O/12**.

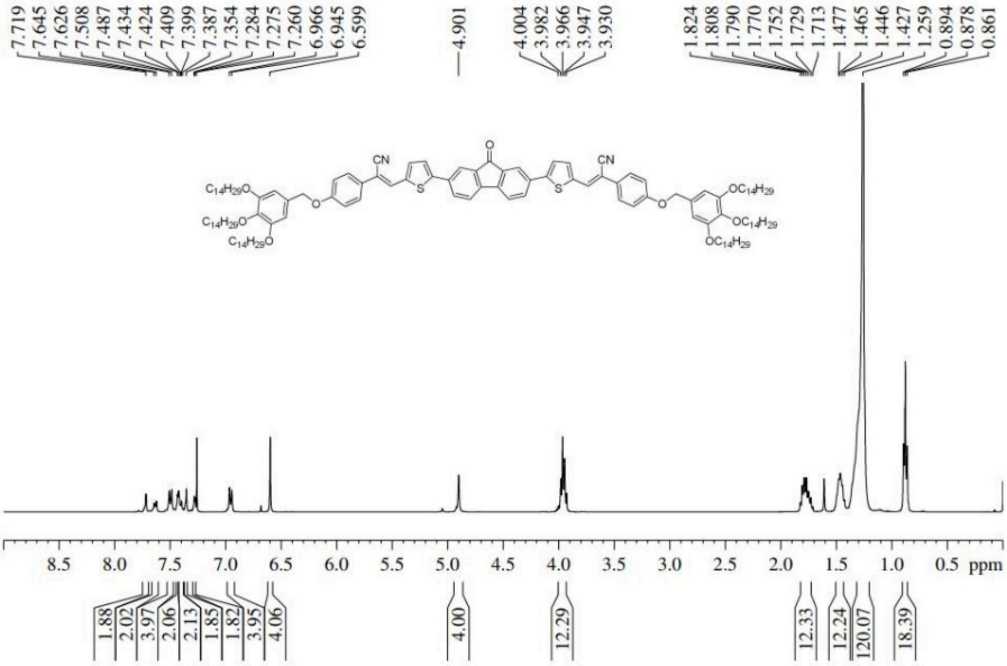

Figure S19. ^{1}H NMR ($CDCl_3$, 400 MHz ppm) spectra of **O/14**.

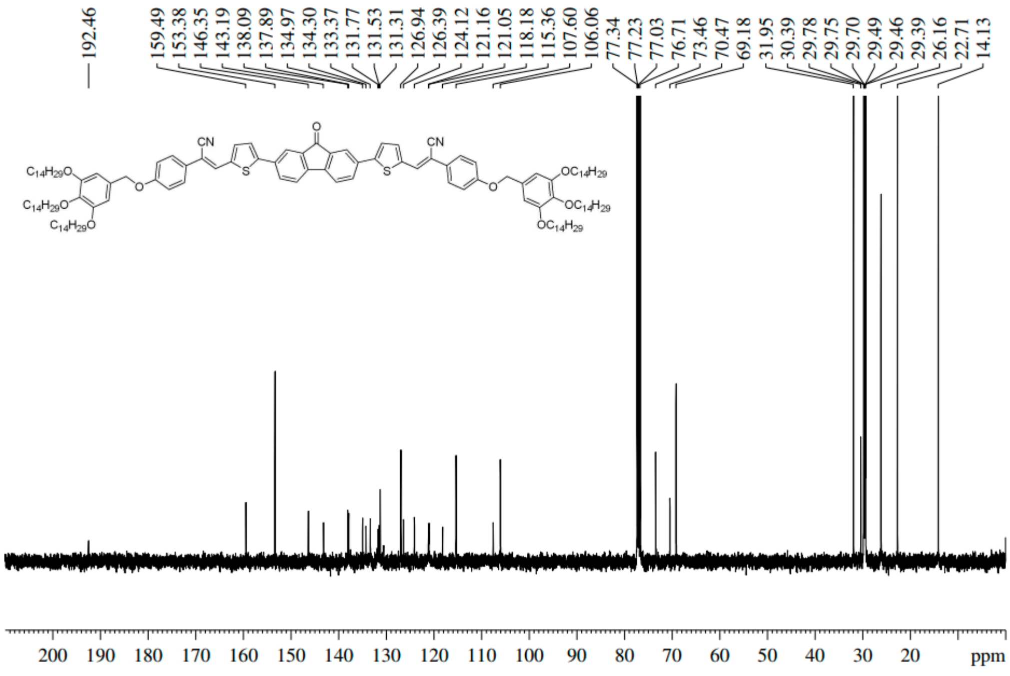

Figure S20. ^{13}C NMR ($CDCl_3$, 100 MHz ppm) spectra of **O/14**.

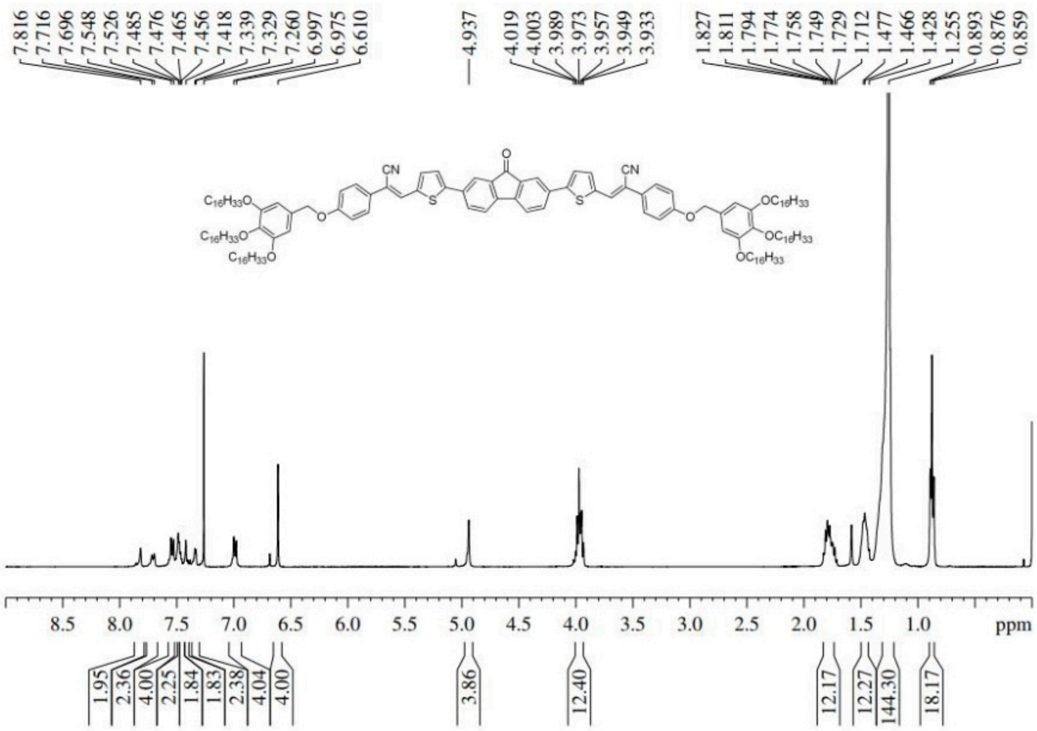

Figure S21. ^{1}H NMR ($CDCl_3$, 400 MHz ppm) spectra of **O/16**.

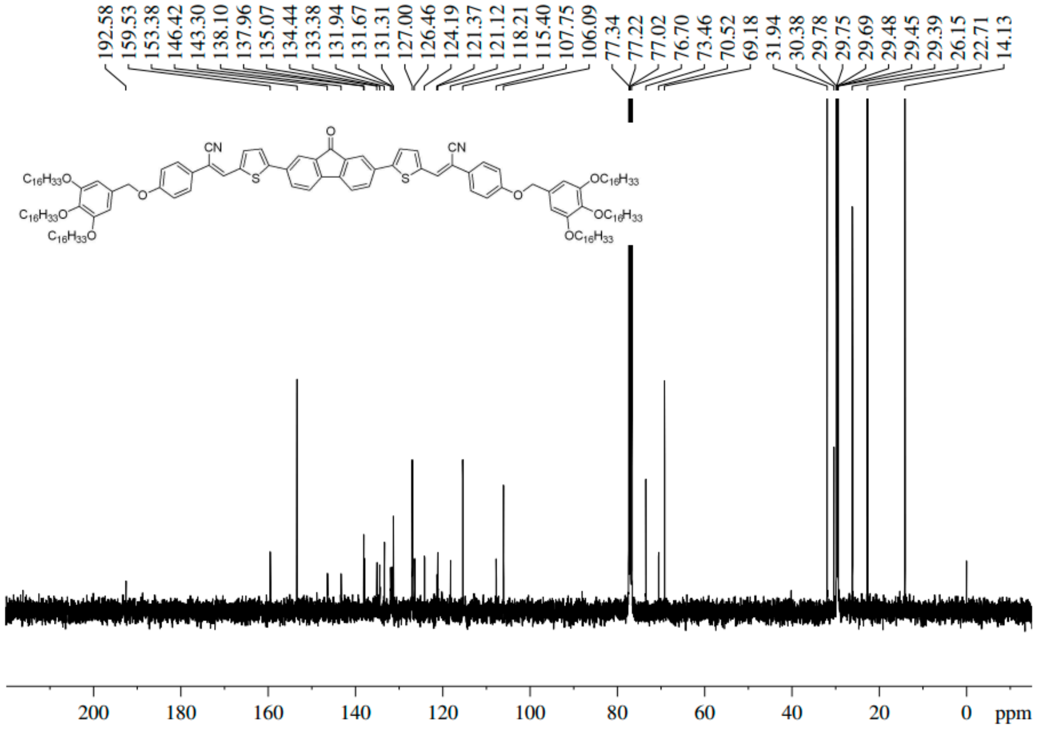

Figure S22. ^{13}C NMR ($CDCl_3$, 100 MHz ppm) spectra of **O/16**.

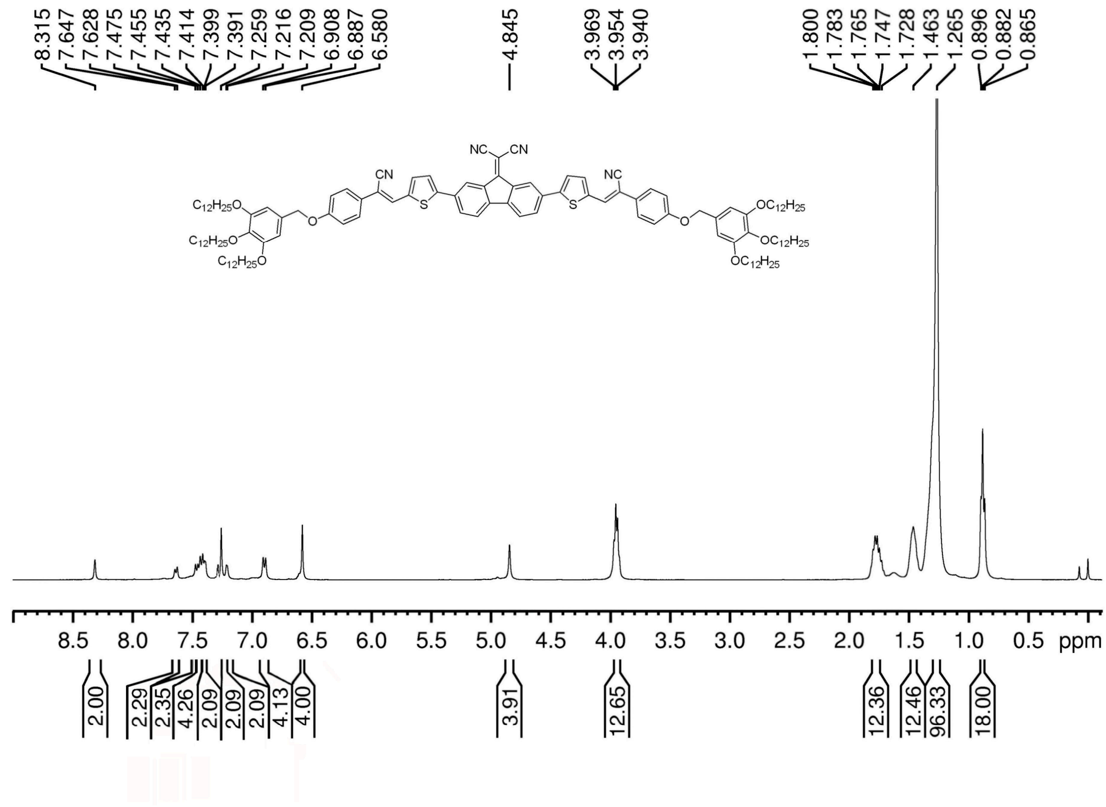

Figure S23. ^{1}H NMR ($CDCl_3$, 400 MHz ppm) spectra of **M/12**.

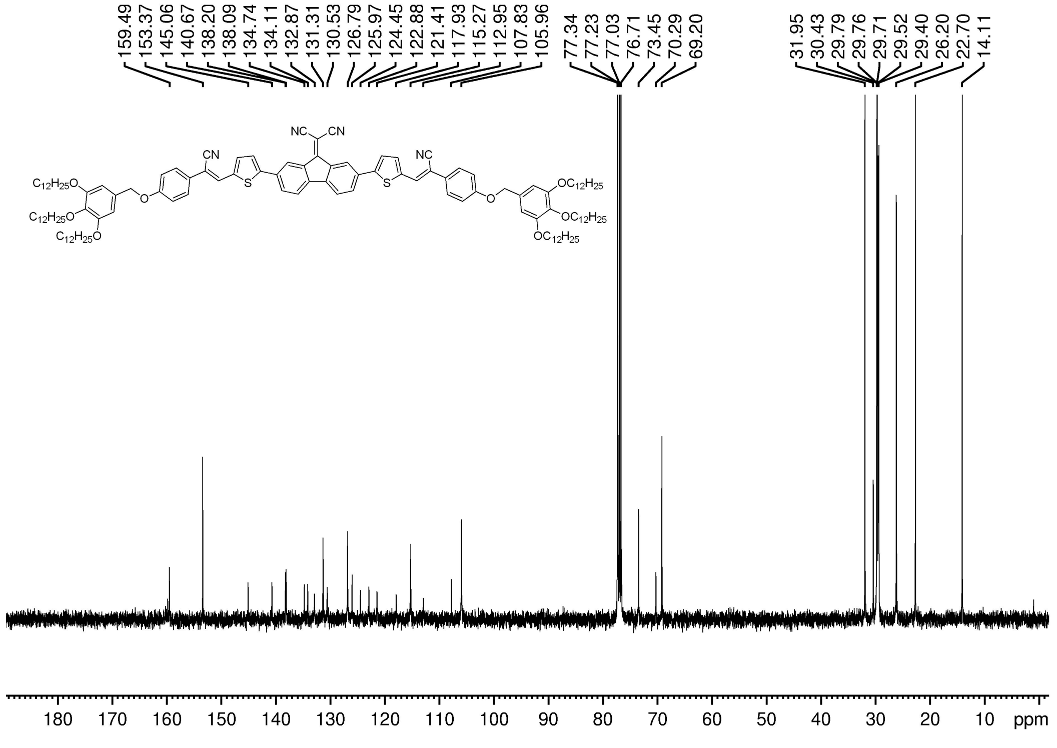

Figure S24. ^{13}C NMR ($CDCl_3$, 100 MHz ppm) spectra of **M/12**.

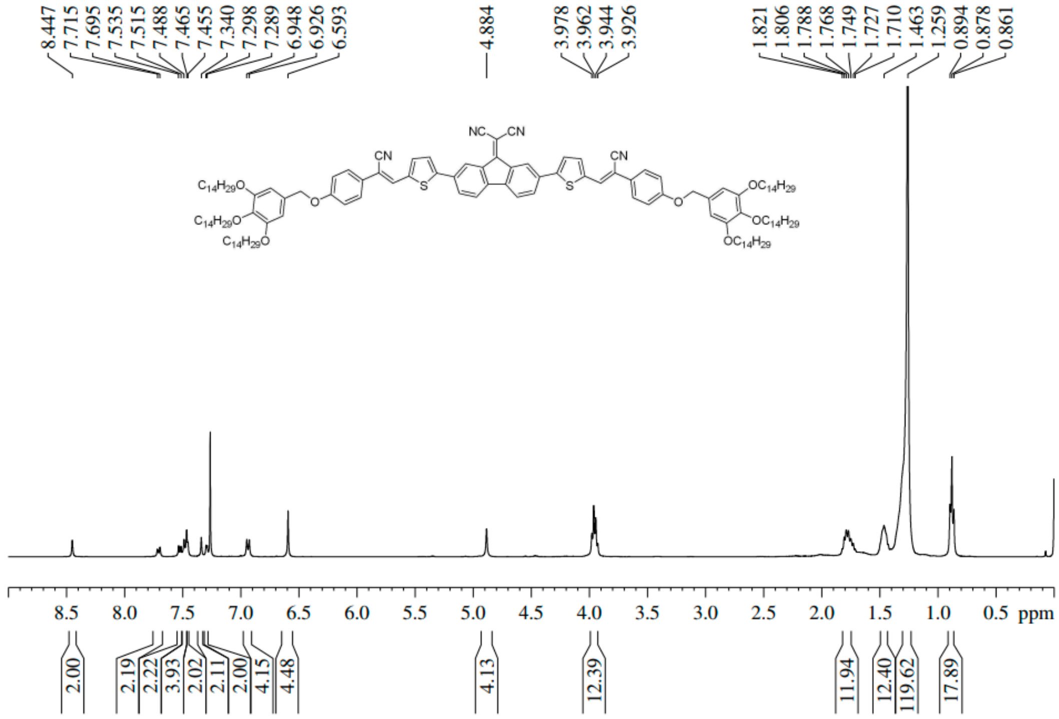

Figure S25. ^{1}H NMR ($CDCl_3$, 400 MHz ppm) spectra of **M/14**.

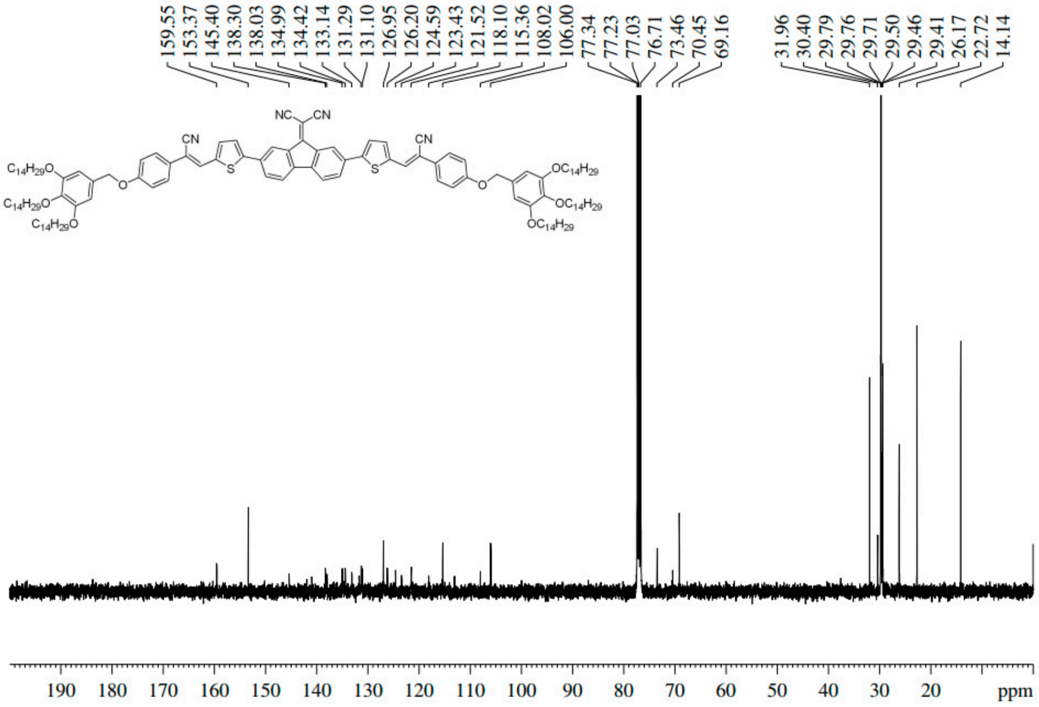

Figure S26. ^{13}C NMR ($CDCl_3$, 100 MHz ppm) spectra of **M/14**.

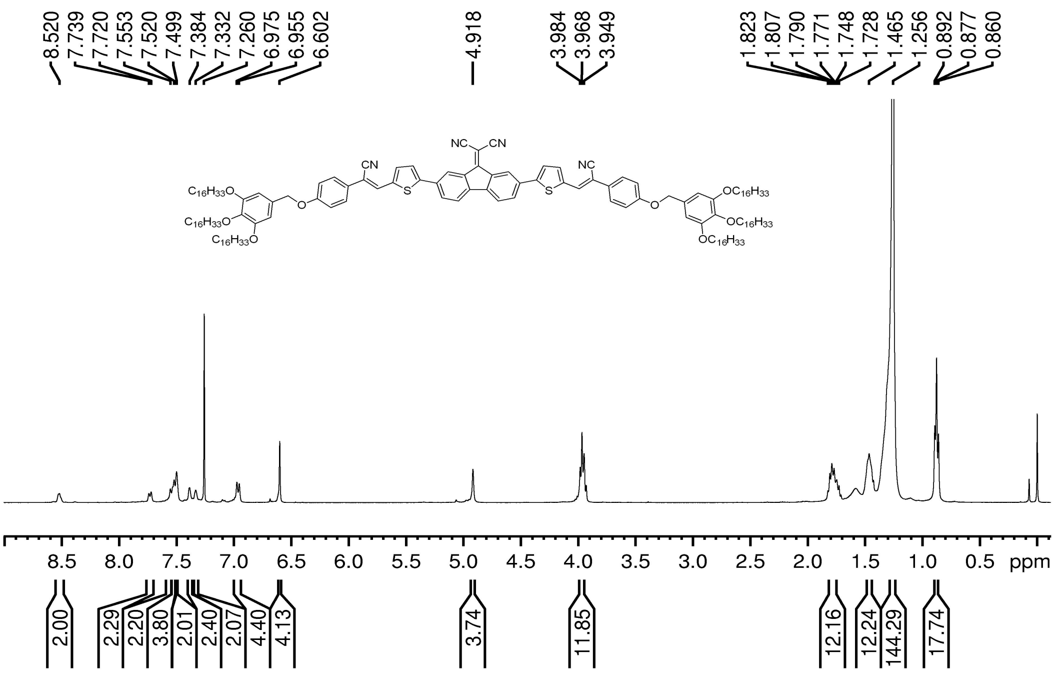

Figure S27. ^{1}H NMR ($CDCl_3$, 400 MHz ppm) spectra of **M/16**.

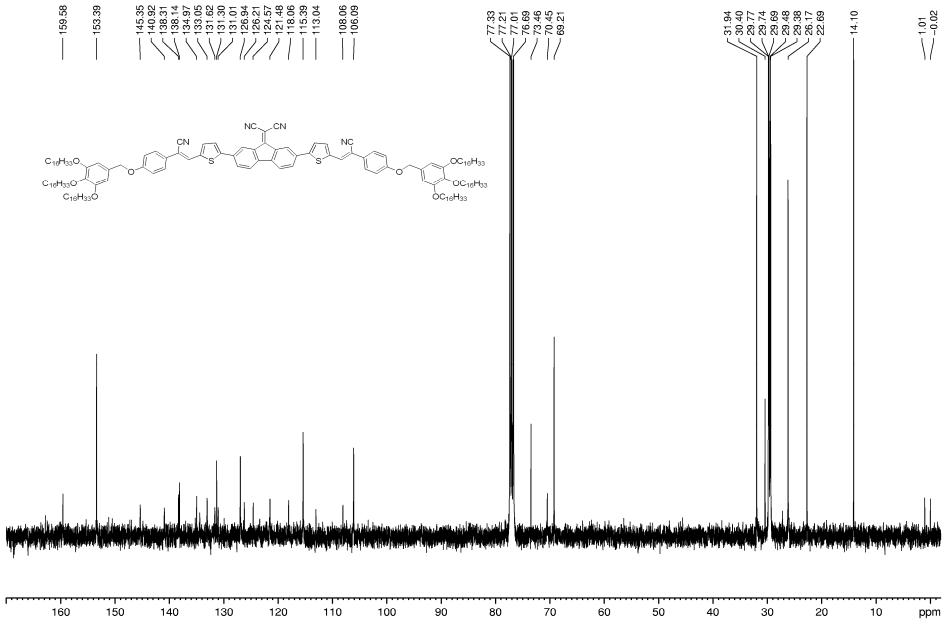

Figure S28. ^{13}C NMR ($CDCl_3$, 100 MHz ppm) spectra of **M/16**.

References

[S1] Li, Z.; Z.,Wu.; Mo,G.; Xing, X.; Liu, P. A SMALL-ANGLE X-RAY SCATTERING STATION AT BEIJING SYNCHROTRON RADIATION FACILITY. *Instrum Sci. Technol* 2014, 42 : 128-141.

[S 2] Williams, A.T.R.; Winfield, S.A.; Miller, J.N. Relative fluorescence quantum yields using a computer-controlled luminescence spectrometer. *Analyst* 1983, 108 : 1067-1071.

[S3] a) Qin, W.; Ding, D.; Liu, J.Z.; Yuan, W.Z.; Hu, Y.; Liu, B.; Tang, B.Z. Biocompatible Nanoparticles with Aggregation-Induced Emission Characteristics as Far-Red/Near-Infrared Fluorescent Bioprobes for In Vitro and In Vivo Imaging Applications. *Adv. Funct. Mater.* 2012, 22 : 771-779; b) Hu, R.R.; Lager, E.; Aguilar-Aguilar, A.; Liu, J.Z.; Lam, J.W.Y.; Sung, H.H.Y.; Williams, I.D.; Zhong, Y.C.; Wong, Y.S.; Pena-Cabrera, E.; Tang, B.Z. Twisted Intramolecular Charge Transfer and Aggregation-Induced Emission of BODIPY Derivatives. *J. Phys. Chem. C* 2009, 113 : 15845-15853.

[S 4] Gobel,D.;. Clamora,N.; Nachtsheim B.J. Regioselective ortho-functionalization of bromofluorenecarbaldehydes using TMPMgCl·LiCl. *Org. Biomol. Chem.* 2018, 16 : 4071-4075.

[S5] Zhang, X.; Ji, X.; Jiang, S.S.; Liu, L.L.; Weeks, B.L.; Zhang, Z. Highly efficient synthesis of 9-fluorenones from 9*H*-fluorenes by air oxidation. *Green Chem.* 2011,13 : 1891-1896.

[S6] Lee, T.; Landis, C.A.; Dhar, B.M.; Jung, B.J.; Sun, J.; Sarjeant,A.; Lee, H,J.; Katz. H. Synthesis, Structural Characterization, and Unusual Field-Effect Behavior of Organic Transistor Semiconductor Oligomers: Inferiority of Oxadiazole Compared with Other Electron-Withdrawing Subunits. *J. Am. Chem. Soc.* 2009, 131: 1692-1705.

[S7] Tan, X.P.; Zhang, R.L.; Guo,C.X.; Cheng, X.H.; Gao, H.F.; Liu, F.; Bruckner, J.R.; Giesselmann, F.; Prehmeand, M.; Tschierske,C. Amphotropic azobenzene derivatives with oligooxyethylene and glycerol based polar groups. *J. Mater. Chem. C* 2015, 3 : 11202-11211; b) Huang, D.X.; Prehm, M.; Gao, H.F.; Cheng, X.H.; Liu, Y.S.; Tschierske, C. Synthesis and self-assembly of luminescent hexacatenar molecules incorporating a 4,7-diphenyl-2,1,3-benzothiadiazole core. *RSC Adv.* 2016, 6 : 21387-21395.

[S8] Zhang, B.; Xiao, Y.L;. Fang, H.P.; Gao, H.F.; Wang, F.K.; Cheng, X.H. Mesogenic D-A fluorophores based on cyanovinyl and benzothiadiazole. *New J. Chem.* 2018, 42 : 16709-16716.
